# Excess success in articles on object-based attention

**DOI:** 10.3758/s13414-022-02459-6

**Published:** 2022-03-01

**Authors:** Gregory Francis, Evelina Thunell

**Affiliations:** 1 Department of Psychological Sciences, Purdue University, West Lafayette, IN USA; 2 École polytechnique fédérale de Lausanne, Brain Mind Institute, Lausanne, Switzerland; 3 Department of Psychology, Stockholm University, Stockholm, Sweden; 4 Department of Clinical Neuroscience, Karolinska Institutet, Stockholm, Sweden

**Keywords:** Attention, Object-based, Statistics

## Abstract

Twenty-five years of research has explored the object-based attention effect using the two-rectangles paradigm and closely related paradigms. While reading this literature, we noticed statistical attributes that are sometimes related to questionable research practices, which can undermine the reported conclusions. To quantify these attributes, we applied the Test for Excess Success (TES) individually to 37 articles that investigate various properties of object-based attention and comprise four or more experiments. A TES analysis estimates the probability that a direct replication of the experiments in a given article with the same sample sizes would have the same success (or better) as the original article. If the probability is low, then readers should be skeptical about the conclusions that are based on those experimental results. We find that 19 of the 37 analyzed articles (51%) seem too good to be true in that they have a replication probability below 0.1. In a new large sample study, we do find evidence for the basic object-based attention effect in the two-rectangles paradigm, which this literature builds on. A power analysis using this data shows that commonly used sample sizes in studies that investigate properties of object-based attention with the two-rectangles paradigm are, in fact, much too small to reliably detect even the basic effect.

**Supplementary Information:**

The online version contains supplementary material available at 10.3758/s13414-022-02459-6.

## Introduction

**Fig. 1:**
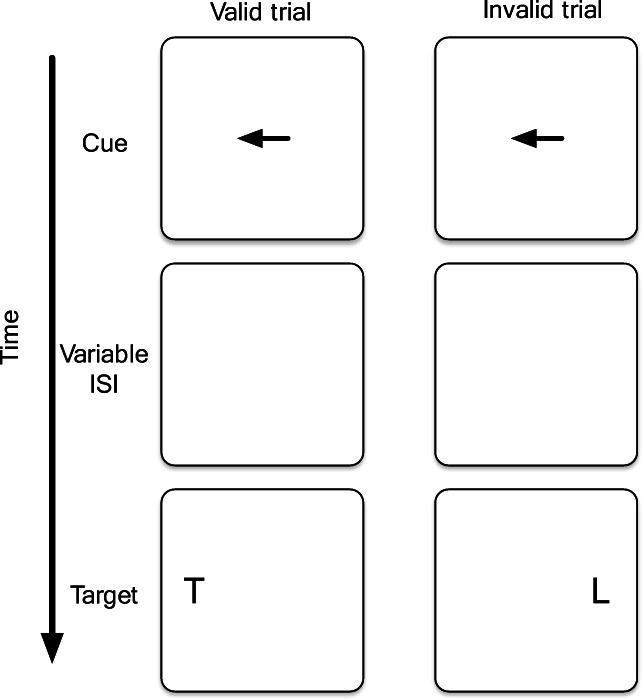
Typical spatial cuing experiment for letter identification. Observers are faster at identifying the target letter in valid (**left**) as compared to invalid (**right**) trials

It is well known that people can process visual information even without directly looking at a stimulus. In particular, we seem to be able to focus processing resources to a certain spatial area, with stimuli outside of this focus taking longer to detect and identify (e.g., Posner, 1980). Such spatial attention effects are well established, and various paradigms have investigated their temporal and spatial properties and limitations. For example, in a spatial cuing task (Fig. 1), a central arrow points to the left or right and indicates with 80% accuracy where a target letter will appear. Observers tend to be about 40 ms faster at identifying a letter appearing at the cued location compared to the uncued location. A neutral condition (with no arrow cue) typically produces a response time between those for the cued and uncued conditions. These results suggest that attentional resources are guided to aid processing at the cued location, and that it takes time to redirect attention from the cued side of the visual field to the uncued side. Other studies have shown that this effect depends on the distance between the target and the cued location (e.g., Mangun & Hillyard, 1988).

**Fig. 2:**
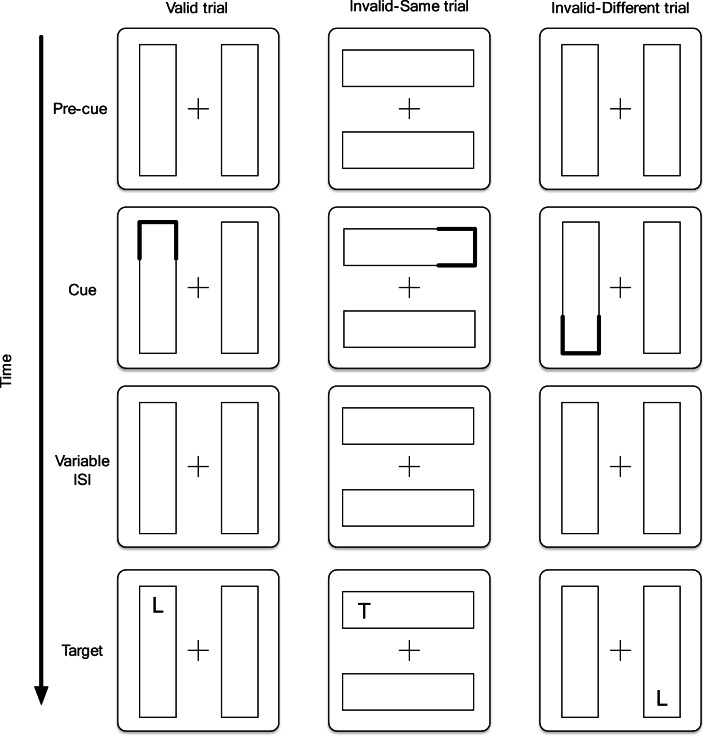
Typical conditions in a two-rectangles study of object-based attention. The key finding is that observers more quickly identify the target letter in the invalid-same than in the invalid-different trials

Visual perception is often concerned with objects rather than with a certain spatial location, and many researchers therefore suspected that attentional processing could have an object-based component. Egly et al. (1994) reported empirical evidence for such object-based attention in a two-rectangles paradigm, schematized in Fig. 2, which has since become the most commonly used paradigm in object-based attention research (Chen, 2012). In this paradigm, two horizontally or vertically aligned rectangles serve as objects. The rectangles are first presented in isolation during a “pre-cue” period. One end of a rectangle is then cued, often by a luminance increment of contour parts. Consistent with spatial cuing effects, response times (e.g., for identifying a subsequent letter as “T” or “L”) are fastest for valid trials, i.e., when the letter appears at the cued location. The invalid trials, i.e., when the letter appears at an uncued location, are divided into two categories of interest: invalid-same and invalid-different. During an invalid-same trial, the target letter appears at the uncued end of the same rectangle as the cue. During an invalid-different trial, the target letter appears at the close end of the rectangle that did not contain the cue. Notably, spatial attention effects should be similar for the invalid-different and the invalid-same trials, because the distance between the target and the cue is the same in both cases. Despite this similarity, Egly et al. (1994) found that response times were faster for the invalid-same as compared to the invalid-different trials. This preferential processing of a target in the cued rectangle is referred to as object-based attention. There are many variations of this paradigm where the objects, the cue, the timing between the cue and the target, the task, and the measurement of performance are modified.

Our interest in object-based attention was piqued because one of the authors (GF) suspected that a neural network model of visual processing (Francis et al., 2017) could explain some object-based attention effects revealed through the two-rectangles paradigm. However, a literature review raised several concerns about published findings and conclusions. First, the reported object-based attention effect, as measured with the two-rectangles paradigm, is quite small (around 15 ms) compared to other effects based on reaction times (e.g., the spatial cuing effect is around 40 ms and the Stroop effect is around 70 ms). A small mean difference typically requires a large sample in order to produce statistical significance, but object-based attention studies often use quite small sample sizes (10–20 observers is common). These studies should therefore have low power and there should accordingly be many non-significant results in articles that comprise multiple studies, even if the effect exists. Contrary to these expectations, virtually none of the multi-study object-based attention articles in this literature reported experimental failures (sometimes non-significant outcomes were reported, but they were used to support theoretical conclusions and thus not treated as failures). Second, although the object-based attention effect has been replicated many times with small sample studies, a large sample (*n* = 120) study by Pilz et al. (2012) found a very small (non-significant) object-based attention effect of 6.5 ms. Roque and Boot (2015) reported similar results, albeit with fewer observers per condition.

It is concerning that the small sample studies (which should have the lowest power) consistently show confirming results while the large sample studies (which should have the highest power) failed to do so. One explanation could be that some small sample studies of object-based attention are subject to publication bias or other questionable research practices. Therefore, we systematically explored the validity of multi-study articles on object-based attention in the two-rectangles paradigm using the Test for Excess Success (TES), as described in the next section.

## The Test for Excess Success

A Test for Excess Success (TES) analysis (Francis, 2012, 2013a; Ioannidis & Trikalinos, 2007) uses the reported data to estimate the success rate for a set of replication experiments. Using this test, we can detect if the sample sizes used for a set of studies are insufficient to reliably demonstrate the claimed effects. Random sampling ensures that experiments will sometimes fail to reject the null hypothesis, even when the alternative hypothesis is true. When experimental results are consistently successful even though the reported data suggest that some experiments should not be successful, the findings appear “too good to be true.” Scientists should be skeptical about the conclusions drawn from such data sets because it seems plausible that the experiments were not run properly, not analyzed properly, or not fully reported (e.g., Simmons et al., 2011). With multiple studies, uniform success should be rare because basing conclusions on multiple tests engenders low power. For example, the probability that a set of four independent experiments, each with a power of 0.8, all produce significant outcomes is only 0.8^4^ = 0.41. Thus, some “failures” should be expected when conducting multiple studies. The TES analysis formalizes this fundamental property of hypothesis testing. Following convention (Begg & Mazumdar, 1994), we concluded excess success if the success rate for a set of studies was below 0.1. Given that desired power for a single experiment is usually at least 0.8, the 0.1 criterion is rather conservative: We suspect that most scientists would not be content with a success rate just above 0.1.

Some people have questioned the validity and applicability of the TES analysis (e.g., Morey, 2013; Simonsohn, 2012). These concerns have been addressed in Francis (2013a, 2013b), and here we summarize this discussion. One raised concern is that the TES analysis does not take into account other non-reported TES analyses that might have been conducted on other articles, and so is itself subject to publication bias and therefore invalid. Such bias might exist, but it does not undermine the conclusions of a given TES analysis for the reported empirical studies in a multi-study publication. Such bias would be problematic if conclusions were inferred for empirical studies that were not part of the analysis. For this reason, we restrict the conclusion of a TES analysis to just the analyzed set of studies and their corresponding conclusions. We focus on findings reported within a single paper (rather than looking across papers) because then we can be confident that the original authors’ conclusions are based on the set of experiments in the analyzed article.

To demonstrate how the TES analysis was implemented, we here describe the details of three of the investigated publications. *R* source code (R core team, 2017) to reproduce all of these calculations is available at the Open Science Framework, and the Online Supplementary Material (OSM) provides details for all investigations. An important characteristic of the analyzed papers is that they not only produced the basic object-based attention effect, but they also measured the impact of different tasks, stimuli, or methods on the effect. The conclusions of these papers depended on the empirical results of both the basic object-based attention effect and the various modifications of the effect.

### TES Example 1

Abrams and Law (2000) reported a total of eight experiments that investigated whether endogenous attention could operate at the object level. Table 1 summarizes the statistics that contributed to the TES analysis and describes the estimated probability that a replication of every study, with the same sample sizes, would produce the same degree of success.

**Table 1:** Statistical properties of the Abrams and Law (2000) experimental findings

	n	Test statistic	Probability of success
Exp. 1	15	*t*(14)=2.7	0.661
Exp. 2	15	*F*(2,28)=3.5	0.605
Exp. 3a	16	*t*(14)=3.8	0.920
Exp. 3b	16	*t*(14)=2.5	0.602
Exp. 4 (null)	10	*F*(1,9)=3.1	0.698
Exp. 5	15	*t*(14)=2.2	0.491
Exp. 6	16	*t*(15)=5.67	0.999
Exp. 7	15	*t*(14)=3.29	0.824
*P*_*TES*_			0.063

Experiment 1 replicated the object-based attention effect reported in Egly et al. (1994) by showing significantly faster reaction times for the uncued (invalid) same-object than the uncued different-object condition of the two-rectangles paradigm. Here, there was an exogenous cue in the form of a brightening of one end of a rectangle. The key statistical result was a difference between these two conditions as demonstrated by a *t*-test, *t*(14) = 2.7, *p* = 0.017. For the TES analysis, we suppose that the experiment properly estimated the population effect, which is characterized as a standardized effect for the difference of dependent means. This supposition reflects the hypothesis that the experiments properly estimated the population effect (e.g., with no QRPs); should the results actually be biased by QRPs, then the estimated effects are almost surely smaller than what is published. We calculated an estimate of the standardized effect using the *t* value and the sample size:

$$ \mathrm{Cohen}\hbox{'}\mathrm{s}\ d=\frac{t}{\sqrt{n}}=\frac{2.7}{\sqrt{15}}=0.697 $$

This calculation tends to overestimate the population standardized effect size, at least for small samples, and an unbiased estimate is computed as (Hedges, 1981):

$$ g=\left(1-\frac{3}{4\left(n-1\right)-1}\right)d=0.659 $$

The next step of the TES analysis uses the estimated population effect to compute the power of a future experiment to produce a significant result with the same sample size. We did this using the *pwr* library in R (Champely et al., 2018), but there are also various on-line alternatives (e.g., Francis, 2018). If the effect is of a magnitude indicated by the empirical data, the power for a two-tailed dependent sample *t*-test on the difference between the invalid-same and invalid-different conditions with *n* = 15 is 0.661. This means that a future study using the same sample size has around a 66% chance of selecting a random sample that produces a significant difference.

Experiment 2 investigated endogenous cuing by using a central cue. The conclusions depended on multiple tests, but we estimated the power based only on the observed interaction between interstimulus interval and object cuing conditions. By focusing on one test, our estimate of the probability of success for a replication study most likely overestimates the actual power, because requiring additional significant outcomes can only reduce power.

Experiment 3 assigned participants to independent endogenous and exogenous cuing conditions. These conditions were separately analyzed, so we treat them as Experiments 3a and 3b.

Experiment 4 used a within-subjects design to investigate endogenous and exogenous cuing conditions. The analysis involved multiple tests, out of which a crucial one was a non-significant interaction between type of cue and target location. Although statisticians emphasize that a null result should not be used as evidence for the null hypothesis, the authors treated the null finding as indicating no difference. Thus, we estimated the probability of success for this experiment as one minus the estimated power of that test, which is the probability of a random sample producing a null result.

Experiments 5, 6, and 7 explored various stimulus and task manipulations to compare the findings reported in Experiments 2–4 against previous reports in the literature (which did not find object-based attention effects for endogenous cuing).

Abrams and Law (2000) reported that each of the studies produced results that supported their theoretical conclusion that object-based attention effects can be produced by purely endogenous cues, which suggests a common representation for exogenous and endogenous effects. If the theory is correct, and the population effects are as estimated by the samples, then the probability of getting eight independent studies like these to produce the desired results is the product of the success probabilities: *P*_*TES*_ = .063. That is, even if the effects are real, the reported data suggest that the sample sizes and estimated effects are so small that there is only around a 6% chance of reproducing the observed uniform success across experiments and tests.

The low probability of success begs the question of how the reported findings could have been generated. Over the past few years, scientists have realized that some standard approaches (now called Questionable Research Practices, QRPs) to data collection, analysis, and reporting can lead to overly successful experimental outcomes, even when there are actually no effects in the population (e.g., Francis, 2012; Simmons et al., 2011). We cannot identify precisely what lies behind the results reported in Abrams and Law (2000), but given the ease with which QRPs can happen even when authors do not intentionally set out to mislead readers (Gelman & Loken, 2014), we advise scientists to be skeptical about the validity of the reported results in Abrams and Law (2000) and the conclusions drawn from those results.

### TES Example 2

Using a modified two-rectangles paradigm (same different judgments of two stimuli that appeared either on opposite ends of a common rectangle or on two separate rectangles), Chen and Cave (2008) reported five experiments that investigated the role of endogenous cuing and positional uncertainty in object-based attention. Table 2 summarizes the hypothesis tests that contributed to the TES analysis and describes the estimated probability that a replication of the studies would produce the same degree of success. Chen and Cave (2008) based their conclusions on multiple findings within each study and also on additional comparisons across studies. We considered all of these comparisons by creating simulated datasets sampled from populations that reflect the means, standard deviations, and correlations reported by Chen and Cave (2008).

**Table 2:** Statistical properties of the Chen and Cave (2008) experimental findings

	n	Test statistic	Probability of success
Exp. 1	14	*t*(13)=3.99	.957
Exp. 2	19	*F*(1,18)=4.50	.485
Exp. 3 (null)	14	*t*(13)=1.69	.655
Exp. 4	14	*t*(13)=3.52	.901
Exp. 5 (null)	14	*t*(13)=0.34	.938
Exps. 1 vs 3	--	Interaction	.625
Exps. 1 vs 4 (null)	--	Main effect	.927
Exps. 1 vs 4 (null)	--	Interaction	.931
Exps. 3 vs 5 (null)	--	Main effect	.622
Exps. 3 vs 5 (null)	--	Interaction	.882
Exps. 4 vs 5	--	Interaction	.651
*P*_*TES*_			.088

Experiment 1 verified a previously reported finding that object-based attention effects can occur when the target is presented in a fixed location. A significant object-based attention effect was found for reaction times. Following the analysis in Chen and Cave (2008), the simulation also compared the results from Experiment 1 with those from Experiments 3 and 4. The correlation for within-subject data was computed using the variance sum law for the given means, variances of scores, and variance of difference scores. The calculations are provided in an Excel file that is available at the Open Science Framework. Each of the 100,000 simulated data sets were subjected to the same analyses used by Chen and Cave (2008), and a data set was considered a “success” only if it satisfied all the tests that they used to support their conclusions. We used the proportion of successful simulated data sets as an estimate of the probability of success for the experiment.

Experiment 2 contrasted a block of trials with valid cues against a block with neutral (uninformative) cues. One of the key results was an object by cue interaction, and the statistic for this test is reported in Table 2.

Experiment 3 shortened the presentation duration of the stimulus, and predicted a non-significant effect of object-based attention. The probability of success is the estimated probability of producing a non-significant result.

To verify the role of endogenous cuing, Experiment 4 used a cue without abrupt onsets. Instead of an informative cue, a non-informative cue appeared and partly disappeared to leave an informative component of the cue. A strong object-based attention effect was found.

Experiment 5 used the same kind of cue as in Experiment 4, but with the short stimulus duration of Experiment 3. As for Experiment 3, the observed null result was used to support the authors’ conclusions.

The individual results of these five experiments have fairly high success probabilities. The probability that five experiments like these would all be successful is the product of their individual values: 0.257. However, the conclusions of Chen and Cave (2008) were based not only on these results, but also on six additional comparisons across experiments. Table 2 lists these additional tests and their associated success probabilities.

The comparison of Experiments 1 and 3 revealed a significant interaction of object and experiment. The comparison of Experiments 1 and 4 found no significant difference between experiments or for the object and experiment interaction. Likewise, the comparison of Experiments 3 and 5 found no significant difference for experiment or for the object and experiment interaction. In contrast, there was a significant interaction of object and experiment for comparison of Experiments 4 and 5.

Chen and Cave (2008) reported that each of the studies and the comparisons between studies produced a pattern of results that supported their theoretical conclusion that object-based attention effects could be produced even when target locations are known with certainty through an endogenous cue, which argues against a search prioritization account of object-based attention. If the theory is correct, and the population effects are as estimated by the samples, then the probability of getting five studies like these to produce the desired pattern is: *P*_*TES*_ = .088. This value is calculated directly from the simulated experiments because the different reported results are not independent and thus the success rate is not simply the product of the success probabilities of the different experiments. Since the *P*_*TES*_ value is below the 0.1 criterion, readers should be skeptical about the validity of the reported experimental results in Chen and Cave (2008) and about the theoretical conclusions derived from those results.

### TES Example 3

It might seem like any set of experiments would be deemed to have excess success, but the TES is actually quite conservative (Francis, 2012). An article that does not appear to have excess success is Marrara and Moore (2003), where the results from five experiments, using the two-rectangles paradigm, supported the conclusion that an object-based effect was not driven by the (commonly used) three-sided cue effectively pointing at the location of a same object target. Table 3 summarizes the statistics that contributed to the TES analysis and describes the estimated probability that a replication of each study would produce the same degree of success.

**Table 3:** Statistical properties of the Marrara and Moore (2003) experimental findings

	n	Test statistic	Probability of success
Exp. 1	19	Multiple tests	.985
Exp. 2	16	Multiple tests	.904
Exp. 3	17	Multiple tests	.810
Exp. 4	17	Multiple tests	.951
Exp. 5	16	Multiple tests	.960
*P*_*TES*_			.657

Each experiment analyzed reaction times using an ANOVA for validity (valid, invalid-same object, invalid-different object) followed by one *t*-test comparing the valid and invalid-same conditions, and one *t*-test comparing the invalid-same and invalid-different conditions. Success always required a spatial cuing effect defined as a significant ANOVA and a significant test for valid versus invalid-same. Success for the remaining test was sometimes defined as a significant outcome (indicating an object-based effect) and sometimes as a non-significant outcome (indicating the absence of an object-based effect, or at least a weak effect). Each experiment’s success for producing all three outcomes was estimated based on simulated experiments with data having the same means, standard deviations, and correlations as the reported data. The correlations were computed from the provided statistics, and the calculations are available at the Open Science Framework. In Table 3 we report the success probability for all results in each experiment.

Experiment 1 showed that the display could produce an object-based attention effect. Experiment 2 replaced each rectangle with a pair of separate squares. Although a cuing effect was found, there was no object-based effect. Experiment 3 replaced the squares with a set of four dots that formed a virtual square. Again, there was a cuing effect, but no object-based effect. Experiment 4 used a full grid of dots, so that there was no impression of separate objects. Again, there was a cuing effect, but no object-based effect. Experiment 5 replaced the rectangles with a set of dots that formed similarly sized virtual rectangles. Both a cuing and an object-based effect was found.

Marrara and Moore (2003) reported that each of the studies produced a pattern of results that supported their theoretical conclusion that the object-based attention effect exists and is not due to the directional nature of the three-sided cue. If the theory is correct, and the population effects are as estimated by the samples, then the probability of getting five experiments like these to produce the desired pattern is: *P*_*TES*_ = .657. This value is calculated by multiplying the success probabilities for the independent experiments. Since the *P*_*TES*_ value is above the 0.1 criterion, it does not warrant readers to be skeptical about the reported experimental results in Marrara and Moore (2003) as they relate to the theoretical conclusions. Of course, this does not rule out that the conclusions could be challenged for other reasons, and some scientists might want an even higher replication success rate.

## Systematic investigation of excess success

What do the conclusions of the TES analyses in the previous section mean? The concern about excess success applies to the conclusions and results of a particular analyzed article. For example, although the findings in Abrams and Law (2000) and in Chen and Cave (2008) seem to have excess success, this does not necessarily imply that there is no object-based attention effect in their studies. It is possible that the basic effect exists, but that the more specific theoretical claims (e.g., about the role of endogenous cuing) are unsupported by the reported empirical results. Moreover, because the TES conclusions are restricted to a given article, other articles by these authors or on this topic do not necessarily have similar problems.

To get a sense of the extent of excess success across multi-study papers investigating object-based attention that use the two-rectangles paradigm or similar paradigms, we applied the TES analysis to all articles as of October 2018 that matched the following criteria: First, on Google scholar we used the search term “rectangular” in articles that cited Egly et al. (1994). Searching for this term seemed to return articles with that term and also articles that used the word “rectangle.” This search mostly reported articles that used a version of the two-rectangles method; however, some articles (roughly 22%) used other methods or stimuli to investigate object-based attention. We included the latter as long as they investigated properties of object-based attention. We restricted our analysis to articles with at least four experiments because the TES analysis becomes more sensitive to the impact of questionable research practices with more reported results (this criterion rules out some influential articles, including Egly et al. (1994), which had only two experiments). Importantly, none of the articles measured only the basic object-based attention effect. Rather, they investigated properties or implications of object-based attention (e.g., trying to identify mechanisms underlying the effect or using various stimuli and tasks to mitigate/enhance the effect). Details of the identification and selection of articles can be found in the OSM. We identified 46 articles that matched our criteria, but were unable to analyze nine of them due to insufficient statistical information or because numerous errors in the reported statistics impeded analysis. Details of the unanalyzed publications can be found in the OSM. Although there are non-human animal studies of object-based attention, none of the articles we found with non-human subjects satisfied all of our selection criteria.

A TES analysis was applied to the results in each of the remaining 37 articles. The details of the analysis of each article can be found in the OSM, and the analysis source code is available at the Open Science Framework. Table 4 summarizes the TES analysis for each article, ordered by *P*_*TES*_ value. Since *P*_*TES*_ is an estimate of the probability that a direct replication of the set of studies with the same sample sizes would be as successful as the original set of studies, higher values are better.

**Table 4:** Test for Excess Success (TES) analysis results for each article on object-based attention with four or more experiments. The *P*_*TES*_ value is an estimate of the probability that a direct replication would produce the same degree of success as the original article. Higher values are better, and articles with *P*_*TES*_ values below 0.1 are interpreted as having excess success

Authors	Title	*P*_*TES*_
Marrara and Moore (2003)	Object-based selection in the two-rectangles method is not an artifact of the three-sided directional cue	0.657
Chen and Huang (2015)	Solving the paradox between same-object advantage and different-object advantage	0.624
Bekkering and Pratt (2004)	Object-based processes in the planning of goal-directed hand movements	0.577
Dodd and Pratt (2005)	Allocating visual attention to grouped objects	0.300
Lavie and Driver (1996)	On the spatial extent of attention in object-based visual selection	0.259
Marrara and Moore (2000)	Role of perceptual organization while attending depth	0.235
Law and Abrams (2002)	Object-based selection within and beyond the focus of spatial attention	0.219
Shomstein and Yantis (2002)	Object-based attention: Sensory modulation or priority setting?	0.202
Nicol et al. (2009)	Object-based perception mediates the effect of exogenous attention on temporal resolution	0.180
Atchley and Kramer (2001)	Object and space-based attentional selection in three-dimensional space	0.168
Richard et al. (2008)	Attentional spreading in object-based attention	0.167
Lamy and Egeth (2002)	Object-based selection: The role of attentional shifts	0.166
Zemel et al. (2002)	Experience-dependent perceptual grouping and object-based attention	0.164
List and Robertson (2007)	Inhibition of return and object-based attentional selection	0.124
Hecht and Vecera (2007)	Attentional selection of complex objects: Joint effects of surface uniformity and part structure	0.119
Ho and Atchley (2009)	Perceptual load modulates object-based attention	0.114
Crundall et al. (2007)	Object-based attention is mediated by collinearity of targets	0.107
Şentürk et al. (2016)	Saccade latency indexes exogenous and endogenous object-based attention	0.102
Luo et al. (2018)	Prioritization to visual objects: Roles of sensory uncertainty	0.098
Schendel et al. (2001)	Objects and their locations in exogenous cuing	0.095
Yin et al. (2018)	Object-based attention on social units: Visual selection of hands performing a social interaction	0.092
Chen and Cave (2008)	Object-based attention with endogenous cuing and positional certainty	0.088
Conci and Müller (2009)	The “beam of darkness”: Spreading of the attentional blink within and between objects	0.087
Yeari and Goldsmith (2010)	Is object-based attention mandatory? Strategic control over mode of attention	0.087
Vecera and Behrmann (1997)	Spatial attention does not require preattentive grouping	0.085
Zhao et al. (2013)	Attentional spreading in object-based attention: The roles of target-object integration and target presentation time	0.080
Shomstein and Johnson (2013)	Shaping attention with reward: Effects of reward on space- and object-based selection	0.074
Chen and O'Neill (2001)	Processing demand modulates the effects of spatial attention on the judged duration of a brief stimulus	0.067
Abrams and Law (2000)	Object-based visual attention with endogenous orienting	0.063
Shomstein and Behrmann (2008)	Object-based attention: Strength of object representation and attentional guidance	0.059
Feldmann-Wüstefeld and Schubö (2013)	Textures shape the attentional focus: Evidence from exogenous and endogenous cueing	0.052
Drummond and Shomstein (2010)	Object-based attention: Shifting or uncertainty?	0.046
Goldsmith and Yeari (2003)	Modulation of object-based attention by spatial focus under endogenous and exogenous orienting	0.040
Nah et al. (2018)	Object width modulates object-based attentional selection	0.036
Smith et al. (2016)	Object-based attentional facilitation and inhibition are neuropsychologically dissociated	0.015
Seifried and Ulrich (2011)	Exogenous visual attention prolongs perceived duration	0.013
de-Wit et al. (2009)	Object-based attention and visual area LO	0.002

A key finding is that the *P*_*TES*_ value falls below the 0.1 criterion for 19 of the 37 articles (51%). Thus, most investigations of object-based attention that are based on the two-rectangles paradigm with four or more experiments have excess success, i.e., they seem too good to be true. While this finding is discouraging, the situation seems to be even worse in general topics in psychology. An investigation of articles with four or more experiments in the journal *Psychological Science* found that 36 of 44 articles (82%) had excess success (Francis, 2014), and a similar investigation of psychology-related articles in the journal *Science* found that 15 out of 18 articles (83%) had excess success (Francis et al., 2014). Still, given the long history of psychophysics and experimental control in investigations of perception and attention, one might have hoped that the field of object-based attention would have fared better than it did in comparison to studies of general topics in psychology.

Given that the empirical results in Pilz et al. (2012) already indicate the object-based attention effect in the two-rectangles paradigm is small (perhaps nonexistent when averaged across the population), what is learned from the TES analyses summarized in Table 4? We feel there are two key lessons. First, despite strong previous empirical evidence that the object-based attention effect is small, studies since 2012 have not incorporated that information into the design of their experiments. Instead, scientists continue to use small sample sizes to measure small effects, which is very inefficient and should produce many “failures.” Second, the specific conclusions in studies with excess success should be considered unfounded for articles that have excess success. Thus, we recommend readers be skeptical of nearly half of the conclusions from this literature.

The TES analysis does not allow us to generalize the findings from articles with four or more experiments to articles with fewer experiments. However, it would be surprising if QRPs were applied exclusively to four or more study articles. Thus, we suspect that much of the literature on object-based attention is affected by publication bias or QRPs. It is difficult to know what went wrong in individual articles; sometimes even for the authors themselves. Regardless of how excess success was produced, it is likely that many studies of object-based attention overestimate the effect and that many published findings are unlikely to replicate in new studies with similar sample sizes. Our results suggest that a large proportion of articles in the field have conclusions that, in as much as they derive from the reported empirical findings, should be considered unfounded.

## Implications of excess success across the field

QRPs can interfere with science’s ability to self-correct. With various QRPs, scientists can (perhaps unintentionally) use a data set to support a favored conclusion or to suppress an unfavored conclusion. Such manipulations can make it difficult for a field to empirically resolve disagreements and to converge on appropriate consensus. Consistent with these concerns, we noted three topics where consensus seems difficult or problematic for properties of object-based attention.

### Neurophysiology of object-based attention

de-Wit et al. (2009) investigated object-based attention in patient D. F., who suffers from visual agnosia due to damage in the lateral occipital area of the ventral stream. Based on results from four experiments that compared D. F. to control participants, de-Wit et al*.* concluded that D. F. did not show object-based attention effects, thereby suggesting that the lateral occipital area mediates form processing.

Smith et al. (2016) also investigated patient D. F., and across four experiments they found evidence that D. F. (and comparative controls) exhibited robust inhibition for both space and objects. Smith et al. (2016) go to some length to reconcile the fact that their conclusions differ from those of de-Wit et al. (2009) by pointing out that their stimuli were dynamic rather than static and that both articles report the absence of an excitatory effect for object-based attention.

It is possible that stimulus and task differences could account for the different results in de-Wit et al. (2009) and Smith et al. (2016). However, our TES analysis shows that neither study makes a good argument for their conclusions, and so it is inappropriate to speculate about why they reach different conclusions. The TES analysis indicates that the findings reported in de-Wit et al. (2009) would only replicate with a probability of *P*_*TES*_ = 0.002, while for Smith et al. (2016) the estimated replication probability is *P*_*TES*_ = 0.015. Thus, neither of these articles can actually answer the questions they set out to investigate, simply because the samples are too small.

### Prioritization versus sensory enhancement

There is a long-running debate on whether mechanisms for object-based attention are primarily about prioritization of where to focus attention or about sensory enhancement of attended elements. Experimental results reported within articles often give very consistent support for one theoretical conclusion, even though different articles draw different conclusions about which mechanism is viable. Studies in Table 4 related to this debate include Luo et al. (2018, *P*_*TES*_ = 0.098), Chen and Cave (2008, *P*_*TES*_ = 0.088), Zhao et al. (2013, *P*_*TES*_ = 0.08), Shomstein and Behrmann (2008, *P*_*TES*_ = 0.059), and Drummond and Shomstein (2010, *P*_*TES*_ = 0.046). Given the excess success of these papers, it is not surprising that they are unable to resolve the debate. The conclusions seem to be based on improper studies and so do not meaningfully contribute to the scientific discussion about possible mechanisms.

### The role of endogenous cuing for object-based attention

While QRPs make it very difficult to resolve empirical debates, they can also artificially entrench ideas that perhaps deserve more nuance. In many experiments, object-based attention is guided by an exogenous cue, as in Fig. 2, but four of the articles listed in Table 4 investigated whether object-based attention effects could be driven by an endogenous cue (Abrams & Law, 2000; Chen & Cave, 2008; Feldmann-Wüstefeld and Schubö, 2013; Goldsmith & Yeari, 2003). These articles consistently found evidence that an endogenous cue could support object-based effects. Such broad consensus across independent investigations with different stimuli and tasks might seem to provide converging evidence that endogenous cuing can promote object-based attention. However, each of these studies has excess success, indicating that the reported results are untrustworthy.

## Is there an object-based attention effect?

The TES analysis results in Table 4 cast doubt on many of the conclusions made in studies of object-based attention effects. Such doubt does not, however, prove that those conclusions are wrong. Likewise, excess success in these studies does not indicate that the object-based attention effect does not exist.

A study that used a much larger sample size than any previous investigation (Pilz et al., 2012) found that only a minority of participants exhibited an object-based attention effect, and that some participants exhibited a reversed effect (faster response times for invalid-different than for invalid-same trials). These effects depended on the orientation of the rectangles, with horizontal rectangles showing the object-based effect and vertical rectangles showing the reversed effect. The impact of rectangle orientation has led some researchers to speculate (e.g., Barnas & Greenberg, 2019) that the presumed effects measured in the two-rectangles paradigm is the relative ease of moving attention across the horizontal and vertical meridians rather than an object-based attention effect per se.

Given the concerns about the conclusions for many of the studies in Table 4, we felt it was important to determine whether there is an object-based attention effect at all. Thus, we ran a large sample on-line study using the two-rectangles paradigm.

### Method

#### Participants

To motivate the sample size, we ran a power analysis that supposed the difference of population means for an object-based attention effect was 15 ms, the population standard deviation for each condition was 100 ms, and the correlation between the invalid-same and invalid-different conditions was 0.8. The mean value of 15 ms is commonly reported in experiments using the two-rectangles paradigm. The standard deviation value of 100 ms and the correlation of 0.8 were based on standard deviations across participants for a variety of on-line experiments that measure reaction times (Francis & Neath, 2015). These values are only meant to generate “ballpark” estimates of effects so that we can identify reasonable sample sizes. If these population attributes are correct, then an experiment with *n* =189 participants will have a power of 0.9 for a two-tailed dependent sample *t*-test (Francis, 2018). Participants were recruited from students at Purdue University, who received course credit for participation. Data were collected during the beginnings of the COVID-19 pandemic, when the university switched to on-line classes. To support students who needed to earn course credit, we extended data collection beyond our initial goal and ended up with *n* = 264 participants. Such a sample should have power of 0.97 to detect the hypothesized object-based effect.

#### Apparatus

The experiment was run on-line through a web browser using Javascript and HTML (full source code for a local version of the experiment is available at the Open Science Framework). Given the on-line nature of the experiment, precise details about a participant’s computer and the experiment setting are unknown. However, the experiment code detected the type of device that the participant used to access the experiment, and only allowed participation using a computer (laptop or desktop) by preventing participation with a smart phone or tablet. Thus, response times to stimuli were recorded with the computer keyboard.

#### Task

During a trial, a target letter (T or L) was presented at one of the corners of a two-rectangles display. The participant identified the target letter by pressing the *h*-key for a T and the *k*-key for an L. The experiment was self-paced, with the participant pressing the *j*-key to initiate each trial.

#### Stimuli

The stimuli and task are shown in Fig. 2. As in other object-based attention studies, a trial started with presentation of a pre-cue fixation cross and two outlined rectangles (either vertical or horizontal) for 1 s. One corner of the rectangles was then cued by thick lines for 200 ms. After offset of the cue, the fixation and rectangles remained visible for a cue-target interstimulus interval (ISI) of 200, 500, 1,000, or 1,500 ms before the target letter (randomly chosen to be a T or an L) appeared at the cued (80% of the time), invalid-same (10% of the time), or invalid-different (10% of the time) location. The target remained visible until the participant made a response. A 500-ms feedback notification was shown to indicate an incorrect classification of the target letter, an early response (faster than 100 ms), or a late response (slower than 3 s). After providing their response and after any feedback, the participant was prompted to start the next trial when ready.

There were a total of 200 trials per participant; half with the rectangles arranged vertically and half with the rectangles arranged horizontally. Cue-target ISI was a between-subjects factor randomly assigned to each participant. The participants were advised that the first 20 trials would be treated as practice, so there were 180 experimental trials.

### Results

**Fig. 3:**
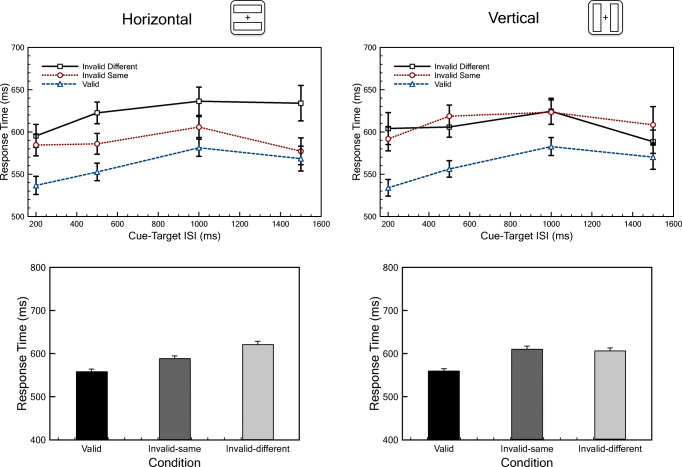
Response time results from the experiment. The top row shows response time as a function of cue-target interstimulus interval (ISI) for the three cue conditions. An object-based attention effect (difference between invalid-different and invalid-same conditions) is present for the horizontal rectangles (**left**) but not for the vertical rectangles (**right**). Error bars indicate standard error of the mean for each data point. The bottom row collapses data across the ISI conditions.

All data and analysis scripts are available at the Open Science Framework. For each participant, we computed mean response times for the three cue conditions (valid, invalid-same, and invalid-different trials). Trials with incorrect responses, early responses, or late responses were not included. Practice trials were also excluded. The top row of Fig. 3 shows the response time for each cue condition as a function of the cue-target ISI. Separate graphs are shown for horizontal and vertical rectangle orientations.

We ran an ANOVA that included cue condition, ISI, and rectangle orientation (with ISI as a between-subjects factor, and condition and orientation as within-subjects factors), and found a main effect of cue condition (*F*(2, 524) = 81.6, *p* < .01) but no main effects of orientation (*F*(1, 262) = 0.67, *p* = .41) or ISI (*F*(1, 262) = 1.52, *p* = .22).

Consistent with other studies, to examine object-based attention effects, we collapsed across (the non-significant main effects of) rectangle orientations and cue-target ISIs. Using a one-way ANOVA, we found a significant effect of cue condition (*F*(2, 526) = 80.6, *p* < .01). Response times were fastest when the target appeared at the cued location $\Big({\overline{X}}_{\mathrm{Cue}}=559$ milliseconds), slower when the target appeared at an uncued location in the same rectangle as the cue $\Big({\overline{X}}_{\mathrm{InvalidSame}}=599$ ms), and slower still when the target was at the uncued location in the non-cued rectangle $\Big({\overline{X}}_{\mathrm{InvalidDifferent}}=613$ ms). The standard deviation of the response times was around 100 ms, in accordance with the assumption in our power calculation, and the correlation between the invalid-same and invalid-different conditions was 0.73; which is somewhat smaller than what was assumed in the power calculation. The object-based cuing effect (the difference in mean response time between the invalid-same and invalid-different conditions) is 14 ms, i.e. very similar to what previous studies have reported, and produced a significant contrast (*t*(526) = 3.15, *p* = .002).

While there was no main effect of rectangle orientation, there was a significant interaction of cue condition and orientation (*F*(2, 524) = 13.9, *p* < 0.01; bottom row of Fig. 3). We ran separate one-way ANOVAs for the different rectangle orientations with subsequent contrasts to compare the invalid-same and invalid-different conditions. Horizontal rectangles produced a strong object-based attention effect (32.6 ms; *t*(526) = 5.55, *p* < .001). Vertical rectangles, however, produced a non-significant *reverse* effect (-4.1 ms, *t*(526) = 0.75, *p* = 0.45). Thus, consistent with Pilz et al. (2012), we find that measures of the object-based effect seem dependent on the orientation of the rectangles. Possibly, there are two effects at play in the two-rectangles paradigm: First, an object-based effect that facilitates attention shifts within objects as opposed to across objects, and second, a vertical/horizontal asymmetry such that horizontal attention shifts are faster than vertical attention shifts. For example, according to Kröse and Julesz (1989), targets on the horizontal meridian are easier to detect than targets on the vertical meridian. This asymmetry may reflect hemifield specificity for some attention processes (Alvarez & Cavanagh, 2005; Chen et al., 2013; Holcombe & Chen, 2012), so that attention can be more efficiently allocated in different hemifields (horizontal rectangles) than within a single hemifield (vertical rectangles). Indeed, Barnas and Greenberg (2016) found more efficient allocation of object-based attention along the horizontal meridian as compared to the vertical meridian. This vertical/horizontal asymmetry effect is typically averaged out in two-rectangle paradigms by using both vertical and horizontal rectangles such that equally many horizontal and vertical shifts constitute trials of the invalid-same and invalid-different conditions. Assuming linearity, the average difference between invalid-same and invalid-different response times over horizontal and vertical rectangles is the true object-based attention effect.

### Questionable analyses

What would be the impact of using Questionable Research Practices (QRPs) with our data? To explore the impact of inappropriate methods for data collection and analysis, we describe an alternate analysis and show how it changes the theoretical conclusions.

Suppose that we were initially convinced that a sample of *n* = 10 should suffice to show an object-based attention effect, since this is a rather typical sample size in the field. We might decide to gather data until a set of ten consecutive participants shows a significant effect. In our data set, this first occurs for participants 7 through 16. That is, we ignore the data from participants 1–6 (perhaps with the ad hoc justification that the data from these participants were pilot data), and we stop data collection after participant 16.

With this small data set, we get a significant one-way ANOVA for cue condition (*F*(2, 18) = 8, *p* = .003), reflecting response times that are fastest for the cued condition $\left({\overline{X}}_{\mathrm{Cue}}=572\kern0.28em \mathrm{ms}\right)$, slower for the invalid-same condition $\left({\overline{X}}_{\mathrm{InvalidSame}}=598\kern0.28em \mathrm{ms}\right)$, and slowest for the invalid-different condition $\left({\overline{X}}_{\mathrm{InvalidDifferent}}=635\kern0.28em \mathrm{ms}\right)$. The standard deviation for these conditions varies from 74 to 113 ms, and the correlation between the two invalid conditions is 0.91. The difference between the invalid conditions is 37 ms (*t*(18) = 2.33, *p* = .03). Thus, our questionable data collection method overestimates both the mean effect and the correlation between conditions. These overestimations go hand in hand with significant results in a small sample, but do not properly represent the values of the larger data set (and presumably of the population). Thus, the result of this QRP is a theoretical conclusion that dramatically inflates the object-based attention effect.

This alternative analysis has only scratched the surface of what can be flexibly applied to the data set. We leave it to the reader to explore what other effects might be produced by other flexible trial exclusion criteria. It is easy to come up with a story to match the results of virtually any questionable analysis (e.g., there is an object-based attention effect for long ISIs but not short ISIs), even though that conclusion does not hold for the full data set.

One fear is that QRPs such as hypothesizing after the results are known (HARKING) are driving some of the conclusions in the object-based attention literature. We do not believe that scientists are deliberately misleading their colleagues or that they knowingly suppress data or findings in a way that biases the interpretations. Rather, we suspect that scientists follow standard practice to justify different types of analyses.

## Conclusions

Roughly half of the multi-study articles on object-based attention effects that meet our inclusion criteria report results that seem too good to be true. The statistics of these articles indicate that, simply due to random sampling, there should be some failed experiments that do not support the article’s conclusions. The absence of such failures is a marker that something has gone wrong in these articles with regard to data collection, analysis, or reporting. We therefore suggest that scientists should be skeptical about those reported results and the corresponding conclusions.

A fundamental problem for many of the studies of object-based attention that we investigated (even those that do not exhibit excess success) is that they seem to be woefully underpowered. A power analysis using optimistic values for the difference of means, standard deviations, and correlation, indicates that an appropriately powered experiment (e.g., 0.8–0.9 power) requires nearly ten times as many participants as is commonly used in the literature. Moving forward, it might be more reasonable to use the findings from our on-line study to guide the design of future experiments. There, we find a difference of means around 14 ms, standard deviations of around 100 ms, and a correlation between invalid-same and invalid-different conditions of around 0.7. If those values are representative of the population, then a new experiment aiming for 90% power needs *n* = 324 observers for a two-tailed *t*-test (Francis, 2018). This analysis is for an experiment aimed at showing just the simplest object-based attention effect. When multiple effects are studied (e.g., differences in object-based attention for exogenous and endogenous cues) even larger samples are needed. In some situations, sufficient power might be achievable with a smaller sample size, as long as the experimental methods increase the difference of means, decrease the standard deviations, or increase the correlation between conditions. Scientists hoping to use smaller sample sizes need to explain why their methods should promote such improvements compared to the data set reported here.^1^ Some scientists may find it useful to preregister their experimental designs (e.g., Munafò et al., 2017) to motivate such considerations.

We suspect that the object-based attention experiments in our analyses have low power because no power analysis was performed. Indeed, none of the studies listed in Table 4 reported a power analysis, even when they directly replicated previous findings. It is common among scientists to use sample sizes similar to previous studies, but if those previous studies generated *p*-values just a bit below the significance criterion, then predicted power for a replication study with the same sample size is barely over 50% (a replication of the experiment is just as likely to be above as below the criterion, assuming the original experiment captured the population effect size). If previous studies used questionable research practices that tend to overestimate effects, then the true power will be even lower.

To end on a positive note, many of the studies in Table 4 have very clever designs, tasks, and stimuli that could answer important questions about perception, attention, and objects. The critical shortcoming for many of these studies is that the sample sizes are so small that the investigations could (should) hardly ever have worked. We conclude that important scientific work can be done by simply adopting the experimental designs from these articles and replicating the experiments with much larger sample sizes. We anticipate that such replication studies will greatly strengthen our understanding of attention and perception.

## Supplementary Information

ESM 1 (DOCX 129 kb)

## Footnotes

1 To some extent, an experiment with more trials per observer can improve power. We show in the OSM that even with a very large number of trials, an experiment needs at least 185 observers to have 90% power to detect the basic object-based attention effect.
