# Supplementary material for: Excess success in articles on object-based attention
Source: Atten Percept Psychophys. 2022 Mar 1;84(3):700–14. doi: 10.3758/s13414-022-02459-6 (PMC8887804; doi:10.3758/s13414-022-02459-6)
Supplement: Supplementary file 1 — (DOCX 129 kb) [file 13414_2022_2459_MOESM1_ESM.docx]

Supplementary material for: Excess Success in Investigations of Object-Based Attention

**Authors:** Gregory Francis^1^ and Evelina Thunell^1,2,3^

**Affiliations:**

^1^Department of Psychological Sciences, Purdue University, USA

^2^Department of Psychology, Stockholm University, Sweden

^3^Department of Clinical Neuroscience, Karolinska Institutet, Sweden

Correspondence to: gfrancis@purdue.edu

**1. Estimating the Probability of Experimental Success**

Success for an experiment often means finding a significant result. The power for one single result from an experiment that uses the same sample size as the original study can be estimated using a simple formula (Champely et al., 2018; Lenth, 2009). However, sometimes more than one significant test result is required for the experiment to be considered successful. In this case, we can use simulations to estimate the probability of a new experiment producing the multiple significant outcomes (power): Simulated data sets that sample from populations having the means, standard deviations, and correlations estimated by the original data are generated and analyzed in the same way as the original data. Success is defined as all relevant tests being significant. When the required information about the original data set is not available, we can instead identify a lowest upper bound of success probability by identifying the weakest single test and computing its power.

Some authors interpret a null result as support for a conclusion of no difference (although this is not proper use of hypothesis testing). In such cases, we define success as the estimated probability of an experiment producing a non-significant result. For a single test result, this probability is simply the complement of the estimated power. For an experiment with multiple outcomes, it is common for authors to require a combination of significant and non-significant outcomes across different comparisons. When possible, we used simulated data sets to estimate the joint success probability across the multiple tests.

Note that the TES analyses estimate success probabilities under the hypothesis that the reported empirical studies are valid, unbiased, and representative of their respective populations. Thus, the TES analyses give a benefit of the doubt to the original studies. Although we sometimes feel that inappropriate analyses were used, we do not make that critique part of the TES analysis for a given article. Likewise, when the original report does not report the sample sizes for different groups, the TES analysis assumes a nearly balanced design, which tends to maximize power.

Success probabilities depend on the significance criterion. Unless the text indicated otherwise, we assumed the standard 0.05 criterion. Sometimes authors claim significance for slightly larger *p* values and we interpreted that to mean that the they used a non-standard (and usually unmentioned) higher significance criterion. We in this case assumed that the used threshold was the smallest number of multiples of 0.005 that was larger than the obtained *p*-value (e.g., 0.055 or 0.06). If authors reported *p* < 0.05, but a recalculation for the given test statistic and degrees of freedom gives *p* > 0.05 (Nuijten et al., 2015), we again interpreted that to mean that the authors used a non-standard significance criterion. Likewise, some theoretical conclusions were based on “marginal” significance, and we interpreted such claims to reflect use of a non-standard significance criterion.

We consider a set of experimental results to have excess success if the probability of replication experiments with the same sample size matching the observed success rate (or better) is less than 0.1. Although this criterion is arbitrary, we consider it to be conservative and that many scientists would be unsatisfied that their results have a less than 10% chance of successful replication if the results are real and similar to what was reported. Excess success indicates that the reported results are unlikely even if the effects exist. We interpret this characteristic as casting skepticism about the set of results and any conclusions that are based on those results. We should, however, note that for some articles some of the reported findings seem very strong, and it could be the case that some parts of the conclusions are valid, even though the reported findings do not adequately support all of the conclusions.

Source code for all analyses is available on the Open Science Framework (OSF) at <https://osf.io/98x7z/?view_only=3fd151766c154b479e478cbbce629d1a>

**2. Selection of Articles for the TES Analyses**

In early October 2018, we searched for multi-experiment articles referencing the classic Egly, Driver and Rafal (1994) paper that inspired modern investigations of object-based attention. Using google scholar, we tried a variety of search terms and found that the following procedure worked well:

1. On google scholar: <https://scholar.google.com/>, search for the title of Egly *et al.,* “[Shifting visual attention between objects and locations: evidence from normal and parietal lesion subjects](http://psycnet.apa.org/fulltext/1994-34191-001.html)”. This should result in the Egly *et al.* (1994) paper showing up as the first entry, with the link “[Cited by 1226](https://scholar.google.fr/scholar?cites=7501317173674693128&as_sdt=2005&sciodt=0,5&hl=en)” – click on it to display a list of the articles that cite Egly *et al*. (The number 1226 will change, as new citing articles are published).
2. Tick the box “Search within citing articles” and then enter: rectangular "study 2" OR "experiment 2" in the search field above.
3. Now, a text like “About 287 results” should appear at the top of the page and all the citing articles will be listed.
4. We excluded 49 dissertations, 2 conference proceedings, 5 books, 48 obviously off topic articles, and 5 “other” (non-article files, links to personal profile pages, retracted article etc.). This left 178 articles for closer inspection.
5. We downloaded PDF copies of the 178 articles along with any supplemental material. By going through each article, we identified 74 articles that had four or more experiments (54 articles had only two experiments, 43 articles had only three experiments, 5 articles had only one experiment, and 2 articles were reviews or modeling papers).
6. Reading the 74 articles with four or more experiments, we identified 28 as being off topic, relative to our investigation of object-based attention.
7. We attempted to analyze each of the remaining 46 articles to test for excess success, but the test could not be applied to nine of them for a variety of reasons. The articles that could not be analyzed are listed in Table S1 along with the reasons why. Further details are available upon request.

We should note that after completing our TES analyses, we discovered some articles that seem to fit our inclusion criterion but were not identified by this procedure. For example, Davis and Holmes (2005) contains five experiments, cites Egly et al. (1994), and includes the word “rectangular” in the text so it should have been included for analysis. However, this article was not identified by google scholar, and we only discovered it from a reviewer’s comment. In order avoid biasing our conclusions, we did not add articles to our analysis list that were not identified with the above method.

**Table S1**: Articles that included four or more studies but that could not be fully analysed because it was not possible to estimate success probabilities for at least four studies.

| **Year** | **Authors** | **Reason for not being fully analysed by TES** |
| --- | --- | --- |
| 1998 | Behrmann, Zemel & Mozer | The paper contains numerous statistical errors and inconsistencies (degrees of freedom that do not match the sample sizes, *p*-values that do match *F*-values, and a reported difference in means that does not match a graph of the means). These errors make it unclear what the statistical values should be and so estimation of power is problematic. |
| 2002 | Mapelli, Cherubini & Umiltà | Key statements of significance in each experiment are presented without statistics. |
| 2005 | Lazareva, Vecera, Levin & Wasserman | Experiment 1 has no inferential statistics, so there are only 3 experiments on which to base the excess success analysis. Also, some *p*-values do not match reported *t* or *F*-values. |
| 2006 | Lazareva, Vecera & Wasserman | Nearly every experiment misreports the conclusions of some hypothesis tests (significance is claimed for *p*-values well above the 0.05 criterion; e.g., three of the four *F* tests on page 1367 for Experiment 1 are described as significant, but have *p* values of 0.18, 0.098, and 0.1. For experiments 2 and 3, *F* values of 5.43 and 6.27 are described as significant, but for df=(1,1) they produce *p*=0.26 and 0.24, respectively). |
| 2009 | Ho & Yeh | Although described as Experiment 1A and 1B (and 2A and 2B and 3A and 3B), the A and B parts are just different conditions in the experiment. Thus, there are really only 3 experiments in this paper. |
| 2011 | Chou & Yeh | Contains statistical inconsistencies that might be due to reported *t*-values actually being *F*-values. Sent an email to the authors, asking for clarification, but received no reply. |
| 2015 | Greenberg, Rosen, Cutrone & Behrmann | Key results are reported with non-specific statistics (e.g. *p* < 0.01). Too little information is provided for the TES analysis to be performed. |
| 2016 | Lou & Proctor | In Experiment 4A, the statistics for the interaction do not agree with the means and standard deviations in Table 1 and the estimated correlation (derived from the *t*-tests). In Experiment 4B, the between-subjects *t*-test has the wrong *df*. These errors make it unclear what the statistical values should be and so estimation of power is problematic. |
| 2017 | Donovan, Pratt & Shomstein | Reported effect sizes and Bayes factors do not match other statistics. Some reported degrees of freedom do not match the sample sizes. These errors make it unclear what the statistical values should be and so estimation of power is problematic. |

The following sections provide details of the TES analysis for each of the 37 articles where the analysis could be applied. Additional material at the Open Science Framework includes the R scripts (R Development Core Team, 2019) used to estimate success probabilities. Some calculations include spreadsheets that estimate correlations between variables in a within-subject design.

**3. Abrams and Law (2000) “Object-based visual attention with endogenous orienting”**

Abrams and Law (2000) reported a total of eight experiments that investigated whether endogenous attention could operate at the object level. Table S2 summarizes the statistics that contributed to the TES analysis and describes the estimated probability that a replication of each study would produce the same degree of success.

Experiment 1 replicated the object-based attention effect reported by Egly *et al.* (1994) by showing a significant difference in reaction time for the uncued same-object and uncued different-object conditions. Here, the cue was an exogenous cue in the form of a brightening of part of a rectangle.

Experiment 2 investigated endogenous cuing by using a central cue. The analysis included multiple tests, but we base our power estimate on just the interaction between interstimulus interval and object cuing conditions.

Experiment 3 assigned participants to independent endogenous and exogenous cuing conditions. These conditions were analyzed separately, so we treat them as Experiments 3a and 3b.

Experiment 4 used a within-subjects design to investigate endogenous and exogenous cuing conditions. The analysis involved multiple tests, but a crucial test was a non-significant interaction between type of cue and target location. Although a null result should not be used as support for no difference, the authors do treat it as indicating no difference. Thus, we estimate the probability of success for this experiment as one minus the estimated power.

**Table S2**. Statistical properties of the Abrams and Law (2000) experimental findings.

|  | **n** | **Test**  **Statistic** | **Probability of Success** |
| --- | --- | --- | --- |
| Exp. 1 | 15 | *t*(14) = 2.7 | .661 |
| Exp. 2 | 15 | *F*(2,28) = 3.5 | .605 |
| Exp. 3a | 16 | *t*(14) = 3.8 | .920 |
| Exp. 3b | 16 | *t*(14) = 2.5 | .602 |
| Exp. 4 (null) | 10 | *F*(1,9) = 3.1 | .698 |
| Exp. 5 | 15 | *t*(14) = 2.2 | .491 |
| Exp. 6 | 16 | *t*(15) = 5.67 | .999 |
| Exp. 7 | 15 | *t*(14) = 3.29 | .824 |
| *P_TES_* |  |  | .063 |

Experiments 5, 6, and 7 explored various stimulus and task manipulations to contrast the findings reported in Experiments 2 - 4 against previous reports in the literature (which did not find object-based attention effects for endogenous cuing).

Abrams and Law (2000) reported that each of the studies produced a pattern of results that supported their theoretical stance. If the theory is correct, and the population effects are as estimated by the samples, an upper limit of the probability of getting eight studies like these to produce the desired pattern is the product of the success probabilities: *P_TES_* = .063. Since this value is less than the 0.1 criterion, readers should be skeptical about the reported experimental results as they relate to the theory.

**4. Atchley and Kramer (2001) “Object and space-based attentional selection in three-dimensional space”**

Atchley and Kramer (2001) reported four experiments that investigated object-based attention across depth and the influence of endogenous and exogenous cues. Table S3 summarizes the statistics that contributed to the TES analysis and describes the estimated probability that a replication of each study would produce the same degree of success.

Among other tests, Experiment 1 reported a barely significant interaction of object location by depth for reaction times. The other tests produced results consistent with the conclusions, so the power of the interaction test can only overestimate the power of the full set of tests.

Experiment 2 includes many significance tests that investigated both reaction time and accuracy. The weakest relevant test compared errors made when targets were at different depths for a cue at the same depth or at a different depth. All of the other tests produced results consistent with the conclusions, so the power of the full set of results can be no higher than the power of this one test.

The weakest relevant test in Experiment 3 was an interaction of object location by depth. The conclusions hinged on many additional tests, so the success probability for the entire set can be no higher than the probability for this interaction test.

Experiment 4 based its conclusions on both reaction time and accuracy. Here, it was not easy to identify which of the many tests were actually relevant for the conclusions. One clearly relevant test was a significant interaction between cue and object for accuracy. The conclusions were also based on several other successful results, so the success probability of the full set of results must be lower than what is listed in Table S3.

Atchley and Kramer (2001) reported that each of the studies produced a pattern of results that supported their theoretical stance. If the theory is correct, and the population effects are as estimated by the samples, the probability of getting four studies like these to produce the desired pattern is, at most, the product of the success probabilities: *P_TES_* = 0.168. Since this value is above the 0.1 criterion, readers do not need to be skeptical about the reported experimental results as they relate to the theory.

**Table S3**. Statistical properties of the Atchley and Kramer (2001) experimental findings.

|  | **n** | **Test**  **Statistic** | **Probability of Success** |
| --- | --- | --- | --- |
| Exp. 1 | 24 | *F*(1,23) = 4.86 | .533 |
| Exp. 2 | 24 | *F*(1,23) = 4.88 | .535 |
| Exp. 3 | 24 | *F*(1,23) = 6.11 | .629 |
| Exp. 4 | 24 | *F*(1,23) = 14.15 | .936 |
| *P_TES_* |  |  | .168 |

**5. Bekkering and Pratt (2004) “Object-based processes in the planning of goal-directed hand movements.”**

Bekkering and Pratt (2004) reported four experiments that investigated the impact of object-based and space-based selection on pointing movements. Table S4 summarizes the statistics that contributed to the TES analysis and describes the estimated probability that a replication of each study would produce the same degree of success.

Experiment 1 reported a significant interaction for number of objects and uncued trials. The other tests produced results consistent with the conclusions, so the power of the interaction test can only overestimate the power of the full set of tests.

The power of Experiment 2 was estimated with 1000 simulated experiments that required significance for three tests (a main effect of an ANOVA and two tests that used Fisher’s Least Significant Difference, LSD, method). The effects in this study were large, and therefore every simulated experiment was a success for all three tests.

Experiment 3 comprised multiple tests, each one with a strong effect. The estimated success probability is 1.0.

Success probability for Experiment 4 was based on 1000 simulated experiments with multiple tests (a main effect for an ANOVA and six LSD tests). Given the within-subjects design, the simulation needed an estimated correlation between scores from different groups. It was not possible to derive the correlation from the available data, so the simulation considered many different possible correlation values, and used the one that maximized the success probability. In this best-case scenario, all of the simulated experiments were uniformly successful.

Bekkering and Pratt (2004) reported that each of the studies produced a pattern of results that supported their theoretical stance. If the theory is correct, and the population effects are as estimated by the samples, an upper limit of the probability of getting four studies like these to produce the desired pattern is the product of the success probabilities: *P_TES_* = 0.577. Since this value is greater than the 0.1 criterion, the reported experimental results seem generally consistent with the theoretical conclusions.

**Table S4**. Statistical properties of the Bekkering and Pratt (2004) findings.

|  | **n** | **Test**  **Statistic** | **Probability of Success** |
| --- | --- | --- | --- |
| Exp. 1 | 8 | *F*(1,7) = 7.92 | .577 |
| Exp. 2 | 10 | Multiple tests | 1.00 |
| Exp. 3 | 11 | Multiple tests | 1.00 |
| Exp. 4 | 8 | Multiple tests | 1.00 |
| *P_TES_* |  |  | .577 |

**6. Chen and Cave (2008) “Object-based attention with endogenous cuing and positional certainty”**

Chen and Cave (2008) reported five experiments that investigated the role of endogenous cuing and positional uncertainty in object-based attention. Table S5 summarizes the hypothesis tests that contributed to the TES analysis and describes the estimated probability that a replication of the studies would produce the same degree of success. Notably, Chen and Cave (2008) based their conclusions both on multiple findings within each study and on additional comparisons across studies. We considered all of these comparisons by creating simulated datasets that reflect the statistics (means, standard deviations, and correlations) reported by Chen and Cave (2008).

Experiment 1 verified a previously reported finding that object-based attention effects can occur when the target is presented in a fixed location. A significant object-based attention effect was found for reaction times. Following the analysis in Chen and Cave (2008), the simulation also compared the results from Experiment 1 against Experiments 3 and 4. For this reason, also the estimated power for the test within Experiment 1 was computed with simulated data. The correlation for within-subject data was estimated using the variance sum law for the given means, variances of scores, and the variance of difference scores. The calculations are provided in an Excel file that is available at the Open Science Framework.

Experiment 2 contrasted two blocks of trials that differed according to whether they used a valid or neutral (uninformative) cue. One of the key results was an object by cue interaction, and the statistics for this test is reported in Table S5. There seems to be a discrepancy between some of the tests and the reported means, standard deviation, and correlation. We picked a single test to set an upper limit on the power estimate for tests involving Experiment 2.

Experiment 3 shortened the presentation duration of the stimulus, which led to a predicted non-significant effect of object-based attention. The probability of success is the estimated probability of producing a non-significant test result.

To verify the role of endogenous cuing, Experiment 4 used a cue without abrupt onsets. Instead of an informative cue appearing, a non-informative cue was presented and part of it disappeared to leave an informative component to the cue. A strong object-based attention effect was found.

Experiment 5 used the same kind of cue as in Experiment 4, but with the short stimulus duration of Experiment 3. As for Experiment 3, the observed null result was used to support the authors’ conclusions.

The individual results of the five experiments have fairly high success probabilities. The probability that five experiments like these would all be successful is the product of their individual values: 0.257. However, the conclusions of Chen and Cave (2008) were based on these results and six additional comparisons across experiments. Table S5 lists these additional tests and their associated success probabilities.

The comparison of Experiments 1 and 3 revealed a significant interaction of object and experiment. The comparison of Experiments 1 and 4 found no significant difference between experiments or for the object and experiment interaction. Likewise, the comparison of Experiments 3 and 5 found no significant difference for experiment or for the object and experiment interaction. In contrast, there was a significant interaction of object and experiment for Experiments 4 and 5.

**Table S5**. Statistical properties of the Chen and Cave (2008) experimental findings.

|  | **n** | **Test**  **Statistic** | **Probability of Success** |
| --- | --- | --- | --- |
| Exp. 1 | 14 | *t*(13) = 3.99 | .957 |
| Exp. 2 | 19 | *F*(1,18) = 4.50 | .485 |
| Exp. 3 (null) | 14 | *t*(13) = 1.69 | .655 |
| Exp. 4 | 14 | t(13) = 3.52 | .901 |
| Exp. 5 (null) | 14 | *t*(13) = 0.34 | .938 |
| Exps. 1 vs 3 | -- | Interaction | .625 |
| Exps. 1 vs 4 (null) | -- | Main effect | .927 |
| Exps. 1 vs 4 (null) | -- | Interaction | .931 |
| Exps. 3 vs 5 (null) | -- | Main effect | .622 |
| Exps. 3 vs 5 (null) | -- | Interaction | .882 |
| Exps. 4 vs 5 | -- | Interaction | .651 |
| *P_TES_* |  |  | .088 |

Chen and Cave (2008) reported that each of the studies and the comparisons between studies produced a pattern of results that supported their theoretical stance. If the theory is correct, and the population effects are as estimated by the samples, the probability of getting five studies like these to produce the desired pattern is *P_TES_* = 0.088. This value is calculated directly from the simulated experiments because the different reported results are not independent and thus it is not appropriate to simply multiply the success probabilities of the comparisons in Table S5. Since the *P_TES_* value is less than the 0.1 criterion, readers should be skeptical about the reported experimental results as they relate to the theory.

**7. Chen and Huang (2015) “Solving the paradox between same-object advantage and different-object advantage”**

Chen and Huang (2015) reported four experiments that compared a traditional same-object advantage (SOA) against a different-object advantage (DOA). Table S6 summarizes the statistics that contributed to the TES analysis and describes the estimated probability that a replication of each study would produce the same degree of success.

Among other findings, Experiment 1 reported that a DOA was found when comparing two identical targets. The conclusions from this experiment were based on multiple tests, but it was not possible to estimate the correlation between dependent measures. Thus, the result in Table S6 is based on just one of the relevant tests. Experiment 2 was similar to Experiment 1, but used different stimuli to find a DOA.

Experiment 3 reported a DOA when using a target identification task rather than the same/different judgment that was used in Experiments 1 and 2.

Experiment 4 was analyzed with many tests, but we used only the comparison of a different-object condition versus a same-object condition (DOA). The probability of success for this test and additional tests must be lower than for this one test alone.

**Table S6**. Statistical properties of the Chen and Huang (2015) experimental findings.

|  | **n** | **Test**  **Statistic** | **Probability of Success** |
| --- | --- | --- | --- |
| Exp. 1 | 17 | *t*(16) = 4.055 | .952 |
| Exp. 2 | 17 | *t*(16) = 2.867 | .727 |
| Exp. 3 | 17 | *t*(16) = 3.872 | .933 |
| Exp. 4 | 17 | *t*(13) = 4.248 | .967 |
| *P_TES_* |  |  | .624 |

Chen and Huang (2015) reported that each of the studies produced a pattern of results that supported their theoretical stance. If the theory is correct, and the population effects are as estimated by the samples, the probability of getting four studies like these to produce the desired pattern is *P_TES_* = 0.624. This value is calculated by multiplying the success probability for the independent experiments. Since the *P_TES_* value is above the 0.1 criterion, readers do not need to be skeptical about the reported experimental results as they relate to the theory. We should note that in addition to showing the DOA, Chen and Huang (2015) also tested for an effect of SOA. Some studies found an effect of SOA and others did not. The variable success for the SOA did not seem to affect the ultimate conclusions of Chen and Huang (2015), except for noting that the SOA seemed smaller than the DOA. Thus, we did not include the tests for SOA in the TES analysis.

**8. Chen and O’Neill (2001) “Processing demand modulates the effects of spatial attention on the judged duration of a brief stimulus”**

Chen and O’Neill (2001) reported four experiments that used single or dual-task designs, and exogenous or endogenous cues, to investigate the impact of attention on duration processing. Table S7 summarizes the statistics that contributed to the TES analysis and describes the estimated probability that a replication of each study would produce the same degree of success.

Experiment 1 included many hypothesis tests. We estimated success probability for the weakest relevant result (a comparison of duration ratings for same object-same location and different object-near location), which surely overestimates the success rate for the entire set of tests. We should note that some tests reported non-significant (or marginal) outcomes, but these findings either did not influence the conclusions drawn by the authors or were interpreted as support for the conclusions.

Experiment 2 was similar to Experiment 1 but with a single task; it required subjects to perform only the duration rating task. The weakest of many tests was a comparison of duration ratings for a different object-far location condition and a same object-same location condition.

Experiments and 3 and 4 were the same as Experiments 1 and 2 but used an endogenous cue rather than an exogenous cue. Each experiment was analyzed with many tests, so we used the weakest result for each experiment. For Experiment 3 this was a test for the duration rating being different between a cued location condition and a same object-different location condition. For Experiment 4 the weakest relevant result was a test for the duration rating being different between a same object-same location condition and a different object-near location condition.

**Table S7**. Statistical properties of the Chen and O’Neill (2001) experimental findings.

|  | **n** | **Test**  **Statistic** | **Probability of Success** |
| --- | --- | --- | --- |
| Exp. 1 | 30 | *t*(29) = 2.06 | .492 |
| Exp. 2 | 26 | *t*(25) = 2.12 | .507 |
| Exp. 3 | 18 | *t*(17) = 2.11 | .477 |
| Exp. 4 | 24 | *t*(23) = 2.29 | .564 |
| *P_TES_* |  |  | .067 |

Chen and O’Neill (2001) reported that each of the studies produced a pattern of results that supported their theoretical stance. If the theory is correct, and the population effects are as estimated by the samples, the probability of getting five studies like these to produce the desired pattern is *P_TES_* = 0.067. This value is calculated by multiplying the success probability for the independent experiments. Since the *P_TES_* value is lower than the 0.1 criterion, readers should be skeptical about the reported experimental results as they relate to the theory. We should note that including more of the tests used by Chen and O’Neill (2001), both within and across experiments, to support their conclusions would only further weaken the success probability of the entire set. Also, they report an additional experiment that supports their conclusions, but without statistical details. This successful extra experiment can only lower the success probability of the set.

**9. Conci and Müller (2009) “The ‘beam of darkness’: Spreading of the attentional blink within and between objects”**

Conci and Müller (2009) reported four experiments to investigate how object-based attention influences the attentional blink. They concluded that the attentional blink spread across object groupings. Table S8 summarizes the statistics that contributed to the TES analysis and describes the estimated probability that a replication of each study would produce the same degree of success.

Experiment 1 included many hypothesis tests with modest effects. We estimated the success probability for the weakest relevant result (a main effect of target 2 location on target 2 identification, which shows an object-based effect), which surely overestimates success for the entire set. We should note that some tests reported non-significant outcomes, but these findings either did not influence the conclusions drawn by the authors or were interpreted as support for the conclusions.

Experiment 2 was similar to Experiment 1 but addressed a potential confound involving the positions of sequential targets in the attentional blink. Experiment 2 handled the confound by allowing for the two targets to possibly be presented at the same location. The weakest of many relevant tests was a main effect of target 2 location.

Experiment 3 investigated whether an object-based attention effect on attentional blink can be produced across occluded parts of an object. The weakest of many tests reported a significant same-object cost for a 260 ms lag between the targets. The text only reports *p* < 0.03 for this test, but we derived the precise *t-value* from the provided means and by using the precisely given *p*-value for a test at another lag to estimate the standard error of the test statistics. The calculations can be found in an Excel file at the Open Science Framework.

Experiment 4 was very similar to Experiment 3, but used different targets in order to allow for direct comparison with previously reported findings. The weakest of many tests was a main effect of the type of local target (same vs. different).

Conci and Müller (2009) reported that each of the studies produced a pattern of results that supported their theoretical stance. If the theory is correct, and the population effects are as estimated by the samples, the probability of getting four studies like these to produce the desired pattern is *P_TES_* = 0.087. This value is calculated by multiplying the success probabilities for the independent experiments. Since the *P_TES_* value is below the 0.1 criterion, readers should be skeptical about the reported experimental results as they relate to the theory. We should note that including more of the tests used by Conci and Müller (2009) to support their conclusions would only weaken the success probability of the entire set.

**Table S8**. Statistical properties of the Conci and Müller (2009) experimental findings.

|  | **n** | **Test**  **Statistic** | **Probability of Success** |
| --- | --- | --- | --- |
| Exp. 1 | 10 | *F*(1,9) = 5.31 | .469 |
| Exp. 2 | 10 | *F*(1,9) = 9.55 | .711 |
| Exp. 3 | 10 | *t*(9) = 2.62 | .570 |
| Exp. 4 | 10 | *F*(1,9) = 5.18 | .460 |
| *P_TES_* |  |  | .087 |

**10. Crundall, Cole, and Galpin (2007) “Object-based attention is mediated by collinearity of targets”**

Crundall *et al.* (2007) reported five experiments investigating how object-based attention is influenced by the collinearity of targets. They concluded that the same-object bias was strongest when targets appeared on the same straight line within an object. Separation by a corner or angle greatly reduced the same-object bias. Table S9 summarizes the statistics that contributed to the TES analysis and describes the estimated probability that a replication of each study would produce the same degree of success.

Experiment 1 included many hypothesis tests. We estimated success probability for the weakest relevant result (an interaction between target parity and target configuration), which surely overestimates success for the entire set. We should note that some tests reported non-significant outcomes, but these findings did not influence the conclusions drawn by the authors.

Experiment 2 was similar to Experiment 1 but used angled chevrons instead of straight lines. The main finding was the absence of a significant target configuration (on the same object or on different objects). The probability of success is estimated as the probability of a replication study producing a non-significant result.

Experiment 3 used Z shapes in order to demonstrate a within-object effect and its absence based on where targets were placed along the lines of the Z shape. The weakest relevant test for the authors’ conclusions was a main effect of parity (whether the targets were the same or different). This effect motivated a discussion in the conclusion section of Crundall *et al.* (2007).

Experiment 4 also used Z-shapes to investigate the impact of a line traversing the fixation point. The conclusions were based on multiple tests, and it was possible to create simulated data that checked on the success for the two relevant tests. These were a significant difference in reaction time for within-line targets compared to within-object targets, and a non-significant difference for within-object targets compared to between-object targets. Success was defined as the combination of these outcomes.

Experiment 5 showed that collinearity of targets could reverse the object bias effect. In the horizontal condition, targets in separate objects (yet horizontally collinear) were responded to faster. This difference supported the claim of a reverse object bias effect.

**Table S9**. Statistical properties of the Crundall *et al.* (2007) experimental findings.

|  | **n** | **Test**  **Statistic** | **Probability of Success** |
| --- | --- | --- | --- |
| Exp. 1 | 17 | *F*(2, 32) = 4.7 | .748 |
| Exp. 2 (null) | 16 | *F*(1,15) = 3.3 | .635 |
| Exp. 3 | 15 | *F*(1,14) = 11.3 | .840 |
| Exp. 4 | 15 | Multiple tests | .423 |
| Exp. 5 | 9 | *t*(8) =2.9 | .633 |
| *P_TES_* |  |  | .107 |

Crundall *et al.* (2007) reported that each of the studies produced a pattern of results that supported their theoretical stance. If the theory is correct, and the population effects are as estimated by the samples, the probability of getting five studies like these to produce the desired pattern is *P_TES_* = 0.107. This value is calculated by multiplying the success probability for the independent experiments. Since the *P_TES_* value is above 0.1, readers do not need to be skeptical about the reported experimental results as they relate to the theory. We should note that including more of the tests used by Crundall *et al.* (2007) to support their conclusions would only weaken the success probability of the entire set.

**11. de-Wit, Kentridge and Milner (2009) “Object-based attention and visual area LO”**

de-Wit *et al.* (2009) reported four experiments to investigate object-based attention in a patient, D. F., who has a form of visual agnosia due to damage in the lateral occipital (LO) area. Each experiment compared the behavior of D. F. to control subjects. de-Wit *et al.* (2009) concluded that patient D. F. had normal spatial orienting but was uninfluenced by object structure. Table S10 summarizes the statistics that contributed to the TES analysis and describes the estimated probability that a replication of each study would produce the same degree of success.

Experiment 1 included many hypothesis tests. Using the classic two rectangles method, a significant (they report *p*=0.051, so we suppose they are using a criterion of 0.055) same-object reaction time advantage was found for the eight control subjects but patient D. F. did not produce a significant difference (based on 125 difference scores). Likewise, a direct comparison of the patient D. F. and the mean control subject difference scores found a significant difference using a Bayesian method (Crawford & Garthwaite, 2007) to compute the *p* value. The same data for patient D. F. was compared to another set of five control subjects from Experiment 4 (which removed the rectangular shapes, so there would be no object effects) and a non-significant result suggested that patient D. F. did not use object-level effects. Success for all of these tests was estimated with simulated experiments that sampled data from populations having means and standard deviations consistent with the statistics reported in de-Wit *et al.* (2009). The success rate is quite low primarily because the method of Crawford and Garthwaite (2007) has extremely low power (this is largely by design in order to mitigate an otherwise inflated Type I error rate when comparing a single subject against a small group). The reported data of D. F. also happened to fall right between the data of control subjects from Experiment 1 and the data from Experiment 4. In a replication, such precise placement is uncommon, so it was common for the simulated data to fail either the significant difference between D. F. and controls in Experiment 1 or the non-significant difference in Experiment 4.

Experiment 2 used a two-item comparison paradigm with targets on rectangles. Control subjects (Experiment 2A) showed a significant difference between reaction times for targets on different objects compared to targets on the same object. Patient D. F., however, did not show a significant difference. No statistics are reported, so we used a most favorable probability of 0.95 (one minus the Type I error rate) for the success probability in Experiment 2B.

Experiment 3 used a Posner cueing paradigm to investigate spatial orienting. Based on 236 samples for each condition, patient D. F. showed a significant difference in reaction time between cued and opposite side targets (Experiment 3A). The control subjects (Experiment 3B) showed a similar difference in their mean reaction times.

**Table S10**. Statistical properties of the de-Wit *et al.* (2009) experimental findings.

|  | **n** | **Test**  **Statistic** | **Probability of Success** |
| --- | --- | --- | --- |
| Exp. 1 and 4 | 8, 1, 6 | Multiple tests | .005 |
| Exp. 2A | 8 | *t*(7)=3.7 | .804 |
| Exp. 2B | -- | null | .950 |
| Exp. 3A | 236, 236 | *t*(470)=2.38 | .660 |
| Exp. 3B | 8 | *t*(7)=5.51 | .986 |
| *P_TES_* |  |  | .002 |

de-Wit *et al.* (2009) reported that each of the studies produced a pattern of results that supported their theoretical stance. If the theory is correct, and the population effects are as estimated by the samples, then the probability of getting four studies like these to produce the desired pattern is *P_TES_* = .002. This value is calculated by multiplying the success probability for the independent experiments. Since the *P_TES_* value is smaller than the .1 criterion, readers should be skeptical about the reported experimental results as they relate to the theory. We should note that including more of the tests used by de-Wit *et al.* (2009) to support their conclusions would only weaken the success probability of the entire set.

**12. Dodd and Pratt (2005) “Allocating visual attention to grouped objects”**

Dodd and Pratt (2005) reported four experiments to investigate how object-based attention and inhibition of return interacted with a perceptual group. Table S11 summarizes the statistics that contributed to the TES analysis and describes the estimated probability that a replication of each study would produce the same degree of success.

Experiment 1 used a display where squares formed one group and circles another group. A prime to one square reduced reaction time to a target that subsequently appeared in another square relative to a target subsequently appearing in a circle.

Experiment 2 also used squares and circles, but jumbled the positions of the squares so that they did not form a cohesive group. Success in this experiment was a non-significant outcome.

Experiment 3 was a conceptual replication of the previous experiments with a number of modifications to eliminate possible confounds. The relevant test reported a significant effect of object shape.

Experiment 4 was very similar to experiment 3, but used a long delay between the cue and target in order to investigate inhibition of return. One relevant result was a non-significant effect of distance.

**Table S11**. Statistical properties of the Dodd and Pratt (2005) experimental findings.

|  | **n** | **Test**  **Statistic** | **Probability of Success** |
| --- | --- | --- | --- |
| Exp. 1 | 8 | *F*(1, 7) = 6.48 | .496 |
| Exp. 2 (null) | 8 | *F*(1, 7) = 2.09 | .800 |
| Exp. 3 | 15 | *F*(1, 14) = 14.93 | .924 |
| Exp. 4 (null) | 15 | *F*(1, 14) = 1.43 | .816 |
| *P_TES_* |  |  | .300 |

Dodd and Pratt (2005) reported that each of the studies produced a pattern of results that supported their theoretical stance. If the theory is correct, and the population effects are as estimated by the samples, the probability of getting four studies like these to produce the desired pattern is *P_TES_* = 0.300. This value is calculated by multiplying the success probability for the independent experiments. Since the *P_TES_* value is above the 0.1 criterion, readers do not need to be skeptical about the reported experimental results as they relate to the theory.

**13. Drummond and Shomstein (2010) “Object-based attention: Shifting or uncertainty?”**

Drummond and Shomstein (2010) reported four experiments to investigate possible mechanisms for object-based attention. They concluded that object-based guidance is based on attentional prioritization rather than a shift of attention. Table S12 summarizes the statistics that contributed to the TES analysis and describes the estimated probability that a replication of each study would produce the same degree of success.

Before describing the experiments and the TES analysis, we mention that the paper statistics seem to have a number of errors. For example, degrees of freedom are often incorrect, relative to the reported sample size(s) and some *p*-values are inconsistent with the reported test statistic (e.g., in experiment 1 “*F*(1,47)=2.786, *p*=.072” should actually be “*F*(1,46)=2.786, *p*=0.102”). Also, findings were sometimes accepted as support for the conclusions when they were “marginal” (e.g., *p*<0.11) and null results were sometimes taken as evidence of no effect. The TES analysis gives the authors the benefit of the doubt by supposing that these interpretations are appropriate.

The statistical analyses in Drummond and Shomstein (2010) were quite complicated, with multiple comparisons between subjects within an experiment, multiple comparisons within subjects in an experiment, and multiple comparisons between subjects across experiments. Given that the conclusions depended on successful outcomes (both significant and non-significant tests) for a wide variety of statistical tests, we estimated the overall success rate with simulated experiments. We extracted means and standard errors from figure plots. (In some cases the text reported means that did not match the corresponding figure; and then we used the value given in the figure.) Using the given sample sizes, we converted the standard errors to standard deviations. Using the means, standard deviations, and within-subject *t* or *F* tests, we computed the correlation between conditions. The detailed calculations are in an Excel spreadsheet that is available at the Open Science Framework.

We wrote an R program to generate simulated data sets that pulled sample values from populations having the means, standard deviations, and correlations defined by the reported samples. For each set of samples, we applied the same 16 statistical tests as Drummond and Shomstein (2010) with the same criteria (e.g., marginal significance, require significance or non-significance). Table S12 reports the statistics for the original tests and the estimated probability of success for a replication study with the same sample size(s). We provide the corrected degrees of freedom for each test, if the original text is incorrect.

In Experiment 1 a cue among a two-rectangles display was always informative about the possible location (one location clockwise of the cue) for a subsequently presented target, which the subject had to identify as being a T or an L. Separate groups of subjects (*n*=14, 15, and 17, respectively) were exposed to different stimulus onset asynchronies between the cue and the target (200, 400, and 600 milliseconds). Reaction times were compared between conditions where the target and cue were on the same or different objects (rectangles). The combination of a significant result for the SOA=200 ms and non-significant results for the SOA=400 and 600 ms conditions lead to the conclusion that object-based effects are driven by attentional prioritization.

Several additional tests for interactions (involving subsets of SOA values) were also used to support the conclusions. These included a marginally significant interaction of SOA and same- versus different-object, two significant interactions that involved only the SOA=200 and either SOA=400 or SOA=600 conditions, and a non-significant interaction that involved SOA=400 and SOA=600.

Experiment 2 used the SOA=400 condition of Experiment 1 and added a valid cue condition. Two statistical tests were relevant. There was a significant difference in reaction time for invalid versus valid trials and there was a non-significant difference for object type. Experiment 3 was the same as Experiment 2, but the researcher monitored eye movements.

Experiment 4 was similar to the previous experiments, but removed the certainty about the location of the target relative to the cue. The tests again found effects of cue validity and object type.

Many of the tests within Experiments 2-4 have quite high probabilities of success (cue validity is a very large effect, and the other test requires a null finding which is easy to get). However, an additional three tests compared findings between experiments, and two of these tests have rather low success probabilities. Significant interactions of object and experiments for Experiments 2 and 4 and for Experiments 3 and 4 were taken as additional support for the conclusions of the study. A non-significant interaction of validity and experiment for Experiments 2 and 4 was also interpreted as support for the conclusions.

**Table S12**. Statistical properties of the Drummond and Shomstein (2010) experimental findings.

|  | **n** | **Test**  **Statistic** | **Probability of Success** |
| --- | --- | --- | --- |
| Exp. 1 (SOA=200) | 14 | *F*(1, 13) = 37.83 | 1.000 |
| Exp. 1 (SOA=400, null) | 15 | *F*(1, 14) < 2.5 | .664 |
| Exp. 1 (SOA=600, null) | 17 | *F*(1, 16) < 2 | .772 |
| Exp. 1 (SOAxObject) | -- | *F*(1, 46) = 2.7786 | .667 |
| Exp. 1 (SOA[200, 400]xObject) | -- | *F*(1, 27) = 6.39 | .693 |
| Exp. 1 (SOA[200, 600]xObject) | -- | *F*(1, 29) = 4.184 | .438 |
| Exp. 1 (SOA[400, 600]xObject, null) | -- | *F*(1, 30) < 1 | .954 |
| Exp. 2 (Cue validity) | 18 | *F*(1,17) = 97.18 | 1.000 |
| Exp. 2 (Object, null) | 18 | *F*(1,17) = 1 | .900 |
| Exp. 3 (Cue validity) | 14 | *F*(1,13) = 37.42 | 1.000 |
| Exp. 3 (Object, null) | 14 | *F*(1,13) < 1 | .938 |
| Exp. 4 (Cue validity) | 17 | *F*(1,16) = 124.082 | 1.000 |
| Exp. 4 (Object) | 17 | *F*(1,16) = 17.877 | .979 |
| Exps. 2 and 4 (Object x Experiment) | -- | *F*(1,33) = 4.833 | .383 |
| Exps. 3 and 4 (Object x Experiment) | -- | *F*(1,31) = 10.147 | .929 |
| Exps. 2 and 4 (Validity x Experiment, null) | -- | *F*(1,31) < 1 | .607 |
| *P_TES_* |  |  | .046 |

The sixteen tests in Table S12 are not independent, so the probability of success for the entire set of tests is not just the product of the individual test success probabilities. Instead, the simulation checked for each of 10,000 simulated experiments how often all sixteen tests were satisfied within a single simulation. The success probability is quite low because so many constraints are placed on the data that it is difficult for a single sample set to satisfy all of them simultaneously.

Drummond and Shomstein (2010) reported that each of the studies produced a pattern of results that supported their theoretical stance. If the theory is correct, and the population effects are as estimated by the samples, the probability of getting four studies like these to produce the desired pattern is *P_TES_* = 0.046. Since the *P_TES_* value is below the 0.1 criterion, readers should be skeptical about the reported experimental results as they relate to the theory.

**14. Feldmann-Wüstefeld and Schubö (2013) “Textures shape attentional focus: Evidence from exogenous and endogenous cueing”**

Feldmann-Wüstefeld and Schubö (2013) reported eight experiments to investigate how spatial cues affected processing of texture in which stimuli were presented. Table S13 summarizes the statistics that contributed to the TES analysis and describes the estimated probability that a replication of each study would produce the same degree of success.

In Experiment 1 subjects identified a target (color of a line segment) that is placed on a textured surface defined by lines of horizontal or vertical line segments. An exogenous cue (diagonally oriented line segment) could either appear at the same location as (valid) or a different location than (invalid) the target. The target could appear on the same (invalid-inside) or a different (invalid-outside) textured surface. The interstimulus interval between the cue and the target varied from 50 to 200 ms. The analysis reported many different tests, and a key result was a set of null contrasts for accuracy of invalid-inside and invalid-outside trials for ISIs greater than 50 ms. For the TES analysis, we used the weakest such test. Including additional successful tests would only lower the probability of success for the experiment.

Experiment 2 was similar to Experiment 1, but used a central endogenous cue with ISIs that ranged from 100 to 600 ms. One of the many relevant results was a null contrast for accuracy of invalid-inside and invalid-outside conditions for most ISI values. For the TES analysis, we used the weakest such test.

Experiment 3 was similar to Experiment 2, but increased the duration of the endogenous cue from 100 to 1000 ms. Again, there were many relevant tests. The weakest test relevant to the conclusions was a significant contrast for invalid-inside and invalid-outside reaction times for ISI=100 ms.

Experiment 4 was very similar to Experiment 1, but placed the target at random positions, so that the cue was uninformative. One of the relevant tests demonstrated a significant difference in accuracy for the invalid-inside and invalid-outside conditions when ISI=50 ms.

Experiment 5 varied the texture arrangements in the cue and search displays. A key result was a significant difference in accuracy between OLD-invalid-inside and NEW-invalid-inside trials for ISI=0 ms.

Experiment 6 explored the role of surface texture and texture border by changing the texture elements between cue and target but leaving the border unchanged. Key results were non-significant differences in accuracy for invalid-inside and invalid-outside conditions for all ISI values. We used the weakest such test result.

Experiment 7 introduced a gradient in orientation across a textured surface. A key result was a significant difference between invalid-inside and invalid-outside accuracies for ISI=50 ms.

Experiment 8 varied border orientation contrast and texture orientation homogeneity. A key result was a significant difference in accuracy for invalid-inside and invalid-outside under conditions of high border contrast.

**Table S13**. Statistical properties of the Feldmann-Wüstefeld and Schubö (2013) experimental findings.

|  | **n** | **Test**  **Statistic** | **Probability of Success** |
| --- | --- | --- | --- |
| Exp. 1 (null) | 16 | *t*(45)=1.56 | .676 |
| Exp. 2 (null) | 16 | *t*(45)=1.26 | .773 |
| Exp. 3 | 16 | *t*(45)=2.50 | .682 |
| Exp. 4 | 16 | *t*(31)=2.79 | .750 |
| Exp. 5 | 16 | *t*(60)=2.35 | .627 |
| Exp. 6 (null) | 16 | *t*(45)=1.31 | .757 |
| Exp. 7 | 16 | *t*(45)=2.46 | .656 |
| Exp. 8 | 16 | *t*(23.6)=2.45 | .621 |
| *P_TES_* |  |  | .052 |

Feldmann-Wüstefeld and Schubö (2013) reported that each of the studies produced a pattern of results that supported their theoretical stance. If the theory is correct, and the population effects are as estimated by the samples, the probability of getting eight studies like these to produce the desired pattern is *P_TES_* = 0.052. This value is calculated by multiplying the success probability for the independent experiments. Since the *P_TES_* value is smaller than the 0.1 criterion, readers should be skeptical about the reported experimental results as they relate to the theory. Note, too, that Feldmann-Wüstefeld and Schubö (2013) based their conclusions on many other tests (both within and between experiments), which can only further decrease the success probability of the entire set.

**15. Goldsmith and Yeari (2003) “Modulation of object-based attention by spatial focus under endogenous and exogenous orienting”**

Goldsmith and Yeari (2003) reported nine experiments to conclude that both endogenous and exogenous cuing can support object-based attention provided that the cues encourage a wide spread of attention rather than focused attention. Table S14 summarizes the statistics that contributed to the TES analysis and describes the estimated probability that a replication of each study would produce the same degree of success.

Experiment 1A used the two-rectangles procedure to replicate previous findings that exogenous cuing yields a same-object advantage in reaction time. Experiment 1B likewise replicated a non-significant result when using an endogenous cue.

Experiment 2A used an endogenous auditory cue (digits) that specified target location. There was a significant same-object advantage in reaction time. Experiment 2B was similar but used high- or low-pitched tones to different ears to identify target location. This experiment also found a same-object advantage for reaction times.

Experiment 3 was similar to Experiment 1B, but used diffuse-attention instructions for the endogenous cue. A significant same-object advantage was found for reaction times.

Experiment 4 combined the conditions of Experiments 1A and 1B into a within-subjects design. A significant same-object advantage was found for reaction times in the endogenous cue condition.

Experiment 5A used the exogenous cue of Experiment 1A but added a go/no go signal that was designed to keep attention focused rather than spread out. The key result was a non-significant result for a test of the same-object advantage. The text did not report a precise test statistic, so we assume that the probability of success is the complement of making a Type I error (which maximizes the success probability estimate).

Experiment 5B was a control for Experiment 5A. Here the go/no go signal was included but was not mentioned to the subjects. A significant same-object advantage was found.

Experiment 6 used the endogenous auditory cue of Experiment 2B and the go/no go signal of Experiment 5A. The key result was a non-significant same-object advantage. Once again, the text did not report a precise test statistic, so the complement of Type I error is used as the probability of success.

**Table S14**. Statistical properties of the Goldsmith and Yeari (2003) experimental findings.

|  | **n** | **Test**  **Statistic** | **Probability of Success** |
| --- | --- | --- | --- |
| Exp. 1A | 20 | *F*(1,19) = 6.65 | .651 |
| Exp. 1B (null) | 20 | *F*(1,19) = 1.43 | .807 |
| Exp. 2A | 20 | *F*(1,19) = 4.92 | .524 |
| Exp. 2B | 20 | *F*(1,19) = 4.68 | .505 |
| Exp. 3 | 19 | *F*(1,18) = 6.12 | .611 |
| Exp. 4 | 12 | *F*(1,11) = 6.15 | .557 |
| Exp. 5A | 15 | *F*(1,14) < 1 | .950 |
| Exp. 5B | 14 | *F*(1,13) = 15.37 | .926 |
| Exp. 6 | 23 | *F*(1,22) < 1 | .950 |
| *P_TES_* |  |  | .040 |

Goldsmith and Yeari (2003) reported that each of the studies produced a pattern of results that supported their theoretical stance. If the theory is correct, and the population effects are as estimated by the samples, then the probability of getting nine studies like these to produce the desired pattern is *P_TES_* = 0.040. This value is calculated by multiplying the success probability for the independent experiments. Since the *P_TES_* value is smaller than the 0.1 criterion, readers should be skeptical about the reported experimental results as they relate to the theory.

**16. Hecht and Vecera (2007) “Attentional selection of complex objects: Joint effects of surface uniformity and part structure”**

Hecht and Vecera (2007) reported five experiments to conclude that attention can only select nonuniform objects when the surface occurs at a part boundary. Table S15 summarizes the statistics that contributed to the TES analysis and describes the estimated probability that a replication of each study would produce the same degree of success.

The conclusions were based on tests within and between experiments. Within each experiment the tests included: an ANOVA of three trial types (valid, invalid-same object, and invalid-different object), a *t*-test comparing valid and invalid-same object trials, a *t*-test comparing valid and invalid-different object trials, and a *t*-test comparing invalid-same and invalid-different object trials. Success for the first three tests corresponded to a significant result, and the tests had very high power. For the last test success was sometimes a significant result (Experiments 1, 3, and 4) and sometimes a non-significant result (Experiments 2 and 5), and it tended to have a moderate success probability.

For each experiment, we used the means, standard deviations, and estimated correlations to generate simulated data that we then subjected to the same analysis used by Hecht and Vecera (2007). The success probability for each experiment was the proportion of simulated experiments that was successful for all tests. Simulation code is available at the OSF.

Experiment 1 used the two-rectangles procedure to replicate previous findings on the presence of an object-based effect for uniform single-part targets. Experiment 2 replicated a previous finding that an object-based effect was not produced with stimuli containing multiple-region objects.

Experiment 3 found the object-based effect when the multi-part objects were defined by adding two minima of curvature points. Experiment 4 found an object-based effect for similar stimuli that also included a color change consistent with the curvature change.

Experiment 5 found that inconsistent placement of the curvature and color changes produced a non-significant object-based effect.

In addition to the tests within each experiment, Hecht and Vecera (2007) further supported their conclusions with an additional six tests that compared object-based effects using data from different experiments. These tests include an ANOVA across all experiments and significant comparisons of Experiments 1 and 2 and Experiments 4 and 5. Non-significant comparisons were found for Experiments 1 and 3, 1 and 4, and 3 and 4.

**Table S15**. Statistical properties of the Hecht and Vecera (2007) experimental findings.

|  | **n** | **Test**  **Statistic** | **Probability of Success** |
| --- | --- | --- | --- |
| Exp. 1 | 20 | Multiple tests | .660 |
| Exp. 2 | 20 | Multiple tests | .818 |
| Exp. 3 | 20 | Multiple tests | .753 |
| Exp. 4 | 20 | Multiple tests | .672 |
| Exp. 5 | 20 | Multiple tests | .940 |
| ANOVA all | -- | *F*(4,95) =3.3 | .815 |
| Exps. 1 and 2 | -- | *t*(38) = 2.5 | .704 |
| Exps. 4 and 5 | -- | *t*(38) < 1 | .461 |
| Exps. 1 and 3 (null) | -- | *t*(38) = 2.0 | .913 |
| Exps. 1 and 4 (null) | -- | *t*(38) < 1 | .942 |
| Exps. 3 and 4 (null) | -- | *t*(38) < 1 | .940 |
| *P_TES_* |  |  | .119 |

Hecht and Vecera (2007) reported that each of the studies produced a pattern of results that supported their theoretical stance. If the theory is correct, and the population effects are as estimated by the samples, then the probability of getting five studies like these to produce the desired pattern is *P_TES_* = .119. This value is calculated by the proportion of simulated experiment sets that all satisfy the required tests. Since the *P_TES_* value is larger than the .1 criterion, readers do not have to be skeptical about the reported experimental results as they relate to the theory.

**17. Ho and Atchley (2009) “Perceptual load modulates object-based attention”**

Ho and Atchley (2009) used the results from six experiments to conclude that object-based attention effects do not spread as well with high perceptual load. The motivation of the studies was to explore the possibility that previous failures to yield data consistent with a sensory enhancement account of object-based attention were due to the high perceptual load of the task. Table S16 summarizes the statistics that contributed to the TES analysis and describes the estimated probability that a replication of each study would produce the same degree of success.

Experiments 1A, 1B, and 1C measured reaction time for various tasks that varied perceptual load. One of the key results in each of these experiments was an interaction between object (same, different) and location (near, far), as an object advantage was found only when the target was near the cue. There were additional tests that examined differences across Experiments 1A-C, but it is not possible to estimate the success rate for these tests simultaneously.

Experiments 2A, 2B, and 2C measured accuracy for the same tasks, but with a method that reduced target presentation time with a following mask. Each experiment produced a number of strong test results, including non-significant interactions, which are consistent with the conclusions. The weakest result for this set of studies was a test for a three-way interaction across the experiments for object, location, and task (experiment).

**Table S16**. Statistical properties of the Ho and Atchley (2009) experimental findings.

|  | **n** | **Test**  **Statistic** | **Probability of Success** |
| --- | --- | --- | --- |
| Exp. 1A | 14 | *F*(1,13) = 7.31 | .653 |
| Exp. 1B | 14 | *F*(1,13) = 4.87 | .485 |
| Exp. 1C | 14 | *F*(1,13) = 5.1 | .503 |
| Exps. 2A, 2B, 2C | 24, 24, 20 | *F*(2,65) = 4.19 | .718 |
| *P_TES_* |  |  | .114 |

Ho and Atchley (2009) reported that each of the studies produced a pattern of results that supported their theoretical stance. If the theory is correct, and the population effects are as estimated by the samples, then the probability of getting six studies like these to produce the desired pattern is *P_TES_* = .114. This value is calculated by multiplying the success probabilities for the independent experiments. Since the *P_TES_* value is larger than the .1 criterion, readers do not have to be skeptical about the reported experimental results as they relate to the theory.

**18. Lamy and Egeth (2002) “Object-based selection: The role of attentional shifts”**

Lamy and Egeth (2002) used the results from four experiments to conclude that the need to shift attention is a critical factor in whether or not object-based effects are observed within grouped uniformly connected regions. Table S17 summarizes the statistics that contributed to the TES analysis and describes the estimated probability that a replication of each study would produce the same degree of success.

Experiment 1 set out to verify the absence of an object-based effect using a task with two comparison targets that could be on the same object or on different objects. The main result was a non-significant test for object type. The article provides insufficient details to compute an effect size, so we give a probability of success that equals the complement of Type I error.

Experiment 2 varied the stimulus onset asynchrony (SOA) between a cue and target onset. Key findings were that increasing SOA affected reaction time differences for valid vs. invalid cues, but did not impact the object-based advantage. The article includes many other statistics that are related to the conclusions but also does not include enough information to evaluate the success probability for more than one test. We estimate an upper limit on the success probability by using just the test for a difference of validity effects for the 100 ms SOA compared to the 200 and 300 ms SOA trials.

Experiment 3 was the same as Experiment 1, except the targets appeared sequentially, with different SOAs, instead of at the same time. One of the main results was a significant object-based effect.

Experiment 4 used both a cue and two targets, one always presented at the cue location. Such an approach would not require a shift of attention, and thereby should not produce an object-based effect. The design sets up a compatibility effect (same vs. different sized targets), and a significant compatibility effect was important for determining that the experimental manipulations worked as intended.

**Table S17**. Statistical properties of the Lamy and Egeth (2002) experimental findings.

|  | **n** | **Test**  **Statistic** | **Probability of Success** |
| --- | --- | --- | --- |
| Exp. 1 | 11 | *F*(1,10) < 1 | .950 |
| Exp. 2 | 12 | *F*(1,11) = 6.98 | .610 |
| Exp. 3 | 10 | *F*(1,9) = 6.62 | .555 |
| Exp. 4 | 10 | *F*(1,9) = 6.00 | .516 |
| *P_TES_* |  |  | .166 |

Lamy and Egeth (2002) reported that each of the studies produced a pattern of results that supported their theoretical stance. If the theory is correct, and the population effects are as estimated by the samples, then the probability of getting eight studies like these to produce the desired pattern is *P_TES_* = .166. This value is calculated by multiplying the success probability for the independent experiments. Since the *P_TES_* value is larger than the .1 criterion, readers do not have to be skeptical about the reported experimental results as they relate to the theory.

**19. Lavie and Driver (1996) “On the spatial extent of attention in object-based visual selection”**

Lavie and Driver (1996) used the results from four experiments to conclude that object-based effects were not strictly due to spatial effects. Table S18 summarizes the statistics that contributed to the TES analysis and describes the estimated probability that a replication of each study would produce the same degree of success.

Experiment 1 had subjects evaluate (same or different) elements (dashes or dots) in gaps on crossing, differently colored, lines. A key comparison was a significant difference of reaction times for object and far conditions. There were many additional tests, but they did not seem relevant to the main conclusions.

Experiment 2 was similar to Experiment 1, but doubled the number of near trials so that targets were equally likely on both sides of the display. The key result was a significant object-based effect found by comparing object and far conditions.

Experiment 3 was the same as Experiment 2, except the dash/dot in a target gap was a common white color. A significant object-based effect was again found by comparing the object and far reaction times.

Experiment 4 was the same as Experiment 3, except that participants were cued to concentrate on just one side of the display. The conclusions were based on many different tests of reaction time and accuracy measures. There was no significant object-based effect (which was interpreted as being due to a narrow focus of attention on just one side of the display), but there were significant spatial effects as indicated by (among other tests), a significant contrast of valid-near and invalid-near accuracy.

**Table S18**. Statistical properties of the Lavie and Driver (1996) experimental findings.

|  | **n** | **Test**  **Statistic** | **Probability of Success** |
| --- | --- | --- | --- |
| Exp. 1 | 10 | *F*(1,9) < 18.5 | .937 |
| Exp. 2 | 12 | *F*(1,11) = 27.6 | .993 |
| Exp. 3 | 13 | *F*(1,12) = 6.0 | .559 |
| Exp. 4 | 20 | *F*(1,19) = 4.6 | .498 |
| *P_TES_* |  |  | .259 |

Lavie and Driver (1996) reported that each of the studies produced a pattern of results that supported their theoretical stance. If the theory is correct, and the population effects are as estimated by the samples, then the probability of getting four studies like these to produce the desired pattern is *P_TES_* = .259. This value is calculated by multiplying the success probability for the independent experiments. Since the *P_TES_* value is larger than the .1 criterion, readers do not have to be skeptical about the reported experimental results as they relate to the theory.

**20. Law and Abrams (2002) “Object-based selection within and beyond the focus of spatial attention”**

Law and Abrams (2002) used the results from five experiments to conclude that object-based effects could occur for both exogenous and endogenous attention. Table S19 summarizes the statistics that contributed to the TES analysis and describes the estimated probability that a replication of each study would produce the same degree of success.

Experiment 1 was a conceptual replication of the Lavie and Driver (1996) study. Key results included significant effects of spatial cuing and an object-effect. The weaker result was a significant difference for the far and object conditions (object-effect).

Experiment 2 was similar to Experiment 1, but a spatial cue preceded the target. Key results included significant effects of spatial cuing and an object-effect. The weaker result was a significant difference for the far and object conditions (object-effect). The text reported that the sample size was *n*=20, but this does not match the reported degrees of freedom for the *t*-test. We assumed that the sample size was actually *n*=21.

Experiment 3 was the same as Experiment 2, except that it used an endogenous cue instead of an exogenous cue. Key results included significant effects of spatial cuing and an object-effect. The weaker result was a significant difference for the far and same bar conditions (object-effect). Here, too, the reported sample size does not match the reported degrees of freedom. We assumed that the sample size was actually larger than what was reported.

Experiment 4 was similar to Experiment 3, but varied the proportion of trials assigned to different conditions. Key results included significant effects of spatial cuing and an object-effect. The weaker result was a significant difference for the far and object conditions (object-effect).

Experiment 5 was also similar to Experiment 3, but shorted the stimulus presentation. Key results included a significant effect of spatial cuing and a non-significant object-effect. The weaker result was the non-significant object-effect.

**Table S19**. Statistical properties of the Law and Abrams (2002) experimental findings.

|  | **n** | **Test**  **Statistic** | **Probability of Success** |
| --- | --- | --- | --- |
| Exp. 1 | 17 | *t*(16) = 2.6 | .643 |
| Exp. 2 | 21 | *t*(20) = 2.156 | .506 |
| Exp. 3 | 22 | *t*(21) = 3.242 | .846 |
| Exp. 4 | 16 | *t*(15) = 3.591 | .889 |
| Exp. 5 (null) | 16 | *t*(15) = 0.76 | .896 |
| *P_TES_* |  |  | .219 |

Law and Abrams (2002) reported that each of the studies produced a pattern of results that supported their theoretical stance. If the theory is correct, and the population effects are as estimated by the samples, then the probability of getting five studies like these to produce the desired pattern is *P_TES_* = .219. This value is calculated by multiplying the success probability for the independent experiments. Since the *P_TES_* value is larger than the .1 criterion, readers do not have to be skeptical about the reported experimental results as they relate to the theory.

**21. List and Robertson (2007) “Inhibition of return and object-based attentional selection”**

List and Robertson (2007) used the results from five experiments to conclude that object-based inhibition of return (IOR) is susceptible to minor design characteristics but space-based IOR is not. Table S20 summarizes the statistics that contributed to the TES analysis and describes the estimated probability that a replication of each study would produce the same degree of success.

Experiment 1 was a direct replication of a previous study that found the inhibitory component of visual attention can be affected by both spatial and object effects. A key result was an object-based IOR.

Experiment 2 was similar to Experiment 1, but used both a short and a long stimulus onset asynchrony (SOA). The analysis involved a large number of tests that looked for spatial cuing and object effects at a variety of SOAs and trials. A key result was a three-way interaction between SOA, cueing, and session half, which suggested that object-based effects varied with experience at the short SOA. Space-based inhibition did not seem to vary with experience.

Experiment 3 was similar to Experiment 2 but used a different set of cues. The key result was the absence of an object-based effect. The article does not provide a specific test statistic, so for the estimated success probability we use the complement of the Type I error rate.

Experiment 4 was similar to Experiment 3, but used another set of cues. The data analysis involved a very large number of tests. The weakest relevant test was a significant interaction of cueing and SOA.

Experiment 5 was similar to Experiment 4, but varied the timings between a peripheral cue and a central cue. Again, the analysis involved many tests. The weakest such test compared reaction times for cued versus between conditions at the long SOA, which indicated a space-based IOR.

**Table S20**. Statistical properties of the List and Robertson (2007) experimental findings.

|  | **n** | **Test**  **Statistic** | **Probability of Success** |
| --- | --- | --- | --- |
| Exp. 1 | 17 | *t*(16)=-2.68 | .669 |
| Exp. 2 | 17 | *F*(1,16)=4.78 | .499 |
| Exp. 3 (null) | 17 | --- | .950 |
| Exp. 4 | 17 | *F*(3,48)=3.79 | .782 |
| Exp. 5 | 17 | *t*(16)=-2.19 | .500 |
| *P_TES_* |  |  | .124 |

List and Robertson (2007) reported that each of the studies produced a pattern of results that supported their theoretical stance. If the theory is correct, and the population effects are as estimated by the samples, then the probability of getting five studies like these to produce the desired pattern is *P_TES_* = .124. This value is calculated by multiplying the success probability for the independent experiments. Since the *P_TES_* value is larger than the .1 criterion, readers do not have to be skeptical about the reported experimental results as they relate to the theory. However, we note that the conclusions depended on many additional tests (both significant and non-significant) within and between experiments. The article does not report enough information to simulate the dependencies between tests, but the additional tests can only decrease the overall success probability.

**22. Luo, Wu, Wang, and Fu (2017) “Prioritization to visual objects: Roles of sensory uncertainty”**

Luo, Wu, Wang, and Fu (2017) used the results from four experiments to conclude that an attentional-prioritization hypothesis based on positional uncertainty cannot account for object-based effects and that a full account needs to include sensory uncertainty. Table S21 summarizes the statistics that contributed to the TES analysis and describes the estimated probability that a replication of each study would produce the same degree of success.

Experiment 1 used a two-rectangles paradigm to identify an isolated target that was followed by a one- or four-item mask. A cue preceded the target. Both space-based and object-based effects were reported, and the role of the mask was important for each effect. The weakest relevant test was for a significant effect of mask type for space-based effects. There were many additional tests that were just barely significant, so the success probability for the entire set of tests is surely lower than what is estimated for just this one test.

Experiment 2 was similar to Experiment 1, but varied the difficulty of the task by making the masks more similar to the target. Subjects were assigned to different rectangle orientations. A key result was a significant interaction between mask and cue validity. Again, there were multiple tests that were just significant, so the probability of success in Table S21 should be considered an overestimate of the full set of tests.

Experiment 3 was similar to Experiment 2 but measured a perceptual threshold duration rather than accuracy. A key result was, again, a significant interaction between validity and mask.

**Table S21**. Statistical properties of the Luo, Wu, Wang, and Fu (2017) experimental findings.

|  | **n** | **Test**  **Statistic** | **Probability of Success** |
| --- | --- | --- | --- |
| Exp. 1 | 19 | *F*(1,18)=4.42 | .478 |
| Exp. 2 | 12, 15 | *F*(2, 50)=3.41 | .615 |
| Exp. 3 | 9, 8 | *F*(2, 32)=3.97 | .670 |
| Exp. 4 | 20 | *F*(1,19)=4.20 | .498 |
| *P_TES_* |  |  | .098 |

Experiment 4 replaced the masks with circles, which should maintain sensory uncertainty but reduce positional uncertainty. The analysis involved many different tests, and marginal results were often considered as support for the conclusions. The text gives conflicting information about the sample size. We assumed it was *n*=20, which matches the reported degrees of freedom. The weakest test was a main effect of number of circles.

Luo, Wu, Wang, and Fu (2017) reported that each of the studies produced a pattern of results that supported their theoretical stance. If the theory is correct, and the population effects are as estimated by the samples, then the probability of getting four studies like these to produce the desired pattern is *P_TES_* = .098. This value is calculated by multiplying the success probability for the independent experiments. Since the *P_TES_* value is smaller than the .1 criterion, readers should be skeptical about the reported experimental results as they relate to the theory. Moreover, we note that the conclusions depended on many additional tests (both significant and non-significant) within and between experiments. The article does not report enough information to simulate the dependencies between tests, but the additional tests can only decrease the overall success probability; so the estimated success probability is almost surely an overestimate.

**23. Marrara and Moore (2000) “Role of perceptual organization while attending depth”**

Marrara and Moore (2000) used the results from seven experiments to conclude that attention in depth is determined by the perceptual organization of a display. Table S22 summarizes the statistics that contributed to the TES analysis and describes the estimated probability that a replication of each study would produce the same degree of success.

Experiment 1 replicated a previous study, where two rectangles were presented at different depths (via binocular disparity). Consistent with the previous study, there was a significant cuing effect on reaction time with 2D displays but not with 3D displays. For the success probability analysis, we used just the significant effect for 2D displays.

Experiment 2 was similar to Experiment 1, but varied when depth placeholders were presented. There were multiple tests, with the weakest being a significant cuing effect for 2D displays in the short display condition.

Experiment 3 used a screen of placeholders, which prevented subjects from attending to a particular placeholder. They key result was a significant cuing effect.

Experiment 4 included gaps between the figure-eight placeholders used in Experiment 3, which eliminated attention allocation to a cued set of placeholders. The result was a significant cuing effect.

Experiment 5 discouraged grouping of placeholders by jiggling each placeholder around its location. A significant cuing effect was found.

Experiment 6 included a condition with placeholders across several different depths. A significant cuing effect was found for nonrandom displays.

Experiment 7 included a similar display as Experiment 6, but with color assignments that allowed for perceptual grouping in depth. They weakest relevant result was that random displays showed a significant cuing effect.

**Table S22**. Statistical properties of the Marrara and Moore (2000) experimental findings.

|  | **n** | **Test**  **Statistic** | **Probability of Success** |
| --- | --- | --- | --- |
| Exp. 1 | 15 | *t*(14)=2.74 | .674 |
| Exp. 2 | 19 | *t*(18)=2.38 | .578 |
| Exp. 3 | 20 | *F*(1, 19)=87.95 | 1.000 |
| Exp. 4 | 17 | *F*(1,16)=50.20 | 1.000 |
| Exp. 5 | 15 | *F*(1,14)=34.49 | .999 |
| Exp. 6 | 19 | *t*(18)=5.34 | .998 |
| Exp. 7 | 16 | *t*(15)=2.51 | .606 |
| *P_TES_* |  |  | .235 |

Marrara and Moore (2000) reported that each of the studies produced a pattern of results that supported their theoretical stance. If the theory is correct, and the population effects are as estimated by the samples, then the probability of getting seven studies like these to produce the desired pattern is *P_TES_* = .235. This value is calculated by multiplying the success probability for the independent experiments. Since the *P_TES_* value is larger than the .1 criterion, readers do not need to be skeptical about the reported experimental results as they relate to the theory.

**24. Marrara and Moore (2003) “Object-based selection in the two-rectangles method is not an artefact of the three-sided directional cue”**

Marrara and Moore (2003) used the results from five experiments to conclude that the object-based effect was not driven by the (commonly used) three-sided cue effectively pointing at the location of a same-object target. Table S23 summarizes the statistics that contributed to the TES analysis and describes the estimated probability that a replication of each study would produce the same degree of success.

Each experiment analyzed reaction times using an ANOVA for validity (valid, invalid same-object, invalid-different object) followed by a *t*-test comparing the valid and invalid-same condition and another *t*-test comparing the invalid-same and invalid-different conditions. Success always required a significant ANOVA and a significant test for valid vs. invalid-same. Success for the remaining test was sometimes for a significant outcome (indicating an object-based effect) but sometimes for a non-significant outcome (which was interpreted as indicating the absence of an object-based effect). Each experiment’s success for producing all three outcomes was estimated with a program that ran simulated experiments having the means, standard deviations, and correlations as for the reported data. The correlations were computed from the provided statistics. In Table S23 we report the success probability for all results in the experiment.

Experiment 1 showed that the display could produce an object-based attention effect.

Experiment 2 replaced each rectangle with a pair of separate squares. Although a cuing effect was found, there was no object-based effect.

Experiment 3 replaced the squares with a set of four dots that formed a virtual square. Again, there was a cuing effect, but no object-based effect.

Experiment 4 used a full grid of dots, so that there was no sense of different objects. Again, there was a cuing effect, but no object-based effect.

Experiment 5 replaced the rectangles with a set of dots that formed a similarly sized virtual rectangle. Both cuing and object-based effects were found.

**Table S23**. Statistical properties of the Marrara and Moore (2003) experimental findings.

|  | **n** | **Test**  **Statistic** | **Probability of Success** |
| --- | --- | --- | --- |
| Exp. 1 | 19 | Multiple tests | .985 |
| Exp. 2 (null) | 16 | Multiple tests | .904 |
| Exp. 3 (null) | 17 | Multiple tests | .810 |
| Exp. 4 (null) | 17 | Multiple tests | .951 |
| Exp. 5 | 16 | Multiple tests | .960 |
| *P_TES_* |  |  | .657 |

Marrara and Moore (2003) reported that each of the studies produced a pattern of results that supported their theoretical stance. If the theory is correct, and the population effects are as estimated by the samples, then the probability of getting five studies like these to produce the desired pattern is *P_TES_* = .657. This value is calculated by multiplying the success probability for the independent experiments. Since the *P_TES_* value is larger than the .1 criterion, readers do not need to be skeptical about the reported experimental results as they relate to the theory.

**25. Nah, Neppi-Modona, Strother, Behrmann and Shomstein (2018) “Object width modulates object-based attentional selection”**

Nah et al. (2018) used the results from five experiments to conclude that object-based attentional selection is modulated by object width. Table S24 summarizes the statistics that contributed to the TES analysis and describes the estimated probability that a replication of each study would produce the same degree of success.

Experiment 1 investigated attentional shifts to thick or thin rectangles. The conclusions were based on many tests, the weakest being a comparison of reaction time to identify a target in a thin rectangle compared to a thick rectangle for validly cued targets (a space-based effect). Including the other tests would lead to an even lower probability of success.

Experiment 2 replaced each rectangle with a trapezoid that has a thick and a thin end. Multiple tests lead to the conclusion that the size effect noted in Experiment 1 was driven by the size of the landing point of attention (rather than the size of the starting point). The data from Experiment 2 was also used in combination with findings from Experiment 3b to rule out an effect of crowding. A key Experiment 2 result was a significant effect of size for valid trials, which is the weakest of the Experiment 2 tests and thereby sets a lowest upper limit for success probability. (Note, the article seems to have mistakenly listed the degrees of freedom as *df*=31, but it should be *df*=30.)

Experiment 3a replaced the target identification task with a target detection task. Catch trials were include to prevent aimless responding. As for the previous experiments, the conclusions depended on many tests, the weakest being a main effect of size on invalid cue trials.

Experiment 3b was the same as Experiment 2 except that the trapezoids disappeared at the onset of the target. Again, the conclusions were based on many different tests, the weakest being a significant validity by size interaction for reaction time. Including additional tests would further reduce the probability of success for this experiment.

Experiment 4 tracked eye movements to examine the influence of object size on saccadic movements. A key result was a main effect of size on the precision of the last fixation.

**Table S24**. Statistical properties of the Nah et al. (2018) experimental findings.

|  | **n** | **Test**  **Statistic** | **Probability of Success** |
| --- | --- | --- | --- |
| Exp. 1 | 32 | *t*(31)=2.14 | .525 |
| Exp. 2 | 31 | *t*(30)=2.14 | .524 |
| Exp. 3a | 21 | *F*(1,20)=4.52 | .495 |
| Exp. 3b | 26 | *F*(1,25)=5.00 | .550 |
| Exp. 4 | 16 | *F*(1,15)=4.64 | .481 |
| *P_TES_* |  |  | .036 |

Nah et al. (2018) reported that each of the studies produced a pattern of results that supported their theoretical stance. If the theory is correct, and the population effects are as estimated by the samples, then the probability of getting five studies like these to produce the desired pattern is *P_TES_* = .036. This value is calculated by multiplying the success probability for the independent experiments. Since the *P_TES_* value is smaller than the .1 criterion, readers should be skeptical about the reported experimental results as they relate to the theory.

**26. Nicol, Watter, Gray and Shore (2009) “Object-based perception mediates the effect of exogenous attention on temporal resolution”**

Nicol et al. (2009) used the results from five experiments to conclude that object-related processes are enhanced by feature binding from exogenous attention, which subsequently degrades temporal resolution. Table S25 summarizes the statistics that contributed to the TES analysis and describes the estimated probability that a replication of each study would produce the same degree of success.

Experiment 1 replicated a finding of reduced temporal resolution at cued locations. The study found a significant difference in the just noticeable difference (JND, the minimal temporal interval between a first and second target that supported 75% accuracy at a temporal order judgment) for cued and uncued locations.

Experiment 2 changed the stimuli used for the temporal order judgment. The main result was a non-significant finding for cued and uncued JNDs. We note that there seems to be an inconsistency between the reported means, standard errors, and *t* statistic. Details are in the Excel spreadsheet at the Open Science Framework. To estimate the success probability, we assumed that the reported *t*-value is correct.

Experiment 3 was similar to Experiment 2 but removed the rectangles from the frames displaying the targets. The key result was a strong difference in JND between cued and uncued locations.

Experiment 4 was similar to Experiment 3, but it presented the two targets at the same location. There was, again, a significant difference in JND for the cued and uncued locations.

Experiment 5 used different target stimuli and mixed trials so that they appeared either at the same location or at different locations. Different tests explored trials that varied in target location. The weakest test found a significant difference for targets at different locations. The text reports a *df*=10, but given the reported sample size and removal of outliers, it seems it should be *df*=9.

Nicol et al. (2009) reported that each of the studies produced a pattern of results that supported their theoretical stance. If the theory is correct, and the population effects are as estimated by the samples, then the probability of getting five studies like these to produce the desired pattern is *P_TES_* = .180. This value is calculated by multiplying the success probability for the independent experiments. Since the *P_TES_* value is larger than the .1 criterion, readers do not need to be skeptical about the reported experimental results as they relate to the theory.

**Table S25**. Statistical properties of the Nicol et al. (2009) experimental findings.

|  | **n** | **Test**  **Statistic** | **Probability of Success** |
| --- | --- | --- | --- |
| Exp. 1 | 11 | *t*(10)=3.30 | .784 |
| Exp. 2 (null) | 11 | *t*(10)=0.21 | .946 |
| Exp. 3 | 7 | *t*(6)= -3.79 | .783 |
| Exp. 4 | 10 | *t*(9)=2.91 | .659 |
| Exp. 5 | 10 | *t*(9)=2.31 | .471 |
| *P_TES_* |  |  | .180 |

**27. Richard, Lee, and Vecera (2008) “Attentional spreading in object-based attention”**

Richard et al. (2008) used the results from six experiments to conclude that object-based attention operates via a spreading mechanism. Table S26 summarizes the statistics that contributed to the TES analysis and describes the estimated probability that a replication of each study would produce the same degree of success.

Experiment 1 had subjects report the shape of a “bite” in a central shape, while flanking elements might have compatible or incompatible bites. A key result was a significant interaction of target/flanker compatibility and object condition.

Experiment 2 was the same as Experiment 1 but eye movements were monitored to ensure that they were not responsible for the observed results. Again, a key result was the interaction.

Experiment 3 was similar to the previous experiments but included a condition with neutral flankers. There were several tests, and the weakest was a significant benefit for compatible flankers.

Experiment 4 replicated a previously published study, where a not significant interaction supported the overall conclusions. The reported statistics only indicate that the test was statistic was *F*<1, so we give the largest possible success rate for this study (one minus the Type I error rate).

Experiment 5 used the same stimuli as Experiment 4, but included a time gap between presentation of stimuli. Again, the key result was a non-significant interaction.

Experiment 6 was a control experiment that replaced the gaps from Experiment 1 with colored shapes that appeared to fall on top of black rectangles. The weakest key result was a non-significant object-based effect. We should note that the means reported in the text do not match the values plotted in Figure 6b. The success probability in Table S26 is based on the reported statistic rather than the values of the means.

**Table S26**. Statistical properties of the Richard et al. (2008) experimental findings.

|  | **n** | **Test**  **Statistic** | **Probability of Success** |
| --- | --- | --- | --- |
| Exp. 1 | 20 | *F*(1,19)=6.3 | .628 |
| Exp. 2 | 12 | *F*(1,11)=8.4 | .690 |
| Exp. 3 | 20 | *t*(18)= 2.4 | .585 |
| Exp. 4 (null) | 20 | *F*(1,19)<1 | .950 |
| Exp. 5 (null) | 20 | *F*(1,19)=1.7 | .779 |
| Exp. 6 (null) | 6 | *F*(1,19)=1.1 | .888 |
| *P_TES_* |  |  | .167 |

Richard et al. (2008) reported that each of the studies produced a pattern of results that supported their theoretical stance. If the theory is correct, and the population effects are as estimated by the samples, then the probability of getting six studies like these to produce the desired pattern is *P_TES_* = .167. This value is calculated by multiplying the success probability for the independent experiments. Since the *P_TES_* value is larger than the .1 criterion, readers do not need to be skeptical about the reported experimental results as they relate to the theory.

**28. Schendel, Robertson and Treisman (2001) “Objects and their locations in exogenous cuing”**

Schendel et al. (2001) used the results from four experiments to compare and contrast faciliatory and inhibitory components of location- and object-based mechanisms in exogenous orienting. Table S27 summarizes the statistics that contributed to the TES analysis and describes the estimated probability that a replication of each study would produce the same degree of success.

Experiment 1 used elements of Star of David stimuli that could separately cue a location or an object. The data were analyzed with many relevant tests, and the weakest such test found a significant main effect of object cuing on reaction time. Including other tests would further weaken the estimated power of this experiment.

Experiment 2 was similar to Experiment 1 but included flanking horizontal bars so that there was bilateral offset when changes occurred to the Star of David stimuli. Again, there were many tests, with one of the weakest being a significant interaction between object and location cuing.

Experiment 3 used similar stimuli to explore effects of shape mismatches at different SOAs. One key result was a significant main effect of stimulus onset asynchrony.

Experiment 4 was a control experiment that compared target detection within different stimuli under identical cuing conditions. One of the relevant results was described in footnote 5 as a significant effect of cued locations at the short stimulus onset asynchrony.

**Table S27**. Statistical properties of the Schendel et al. (2001) experimental findings.

|  | **n** | **Test**  **Statistic** | **Probability of Success** |
| --- | --- | --- | --- |
| Exp. 1 | 12 | *F*(1,11)=6.86 | .603 |
| Exp. 2 | 16 | *F*(1,15)=4.80 | .494 |
| Exp. 3 | 10 | *F*(1,9)=5.63 | .491 |
| Exp. 4 | 9 | *F*(1,16)=.22 | .649 |
| *P_TES_* |  |  | .095 |

Schendel et al. (2001) reported that each of the studies produced a pattern of results that supported their theoretical stance. If the theory is correct, and the population effects are as estimated by the samples, then the probability of getting four studies like these to produce the desired pattern is *P_TES_* = .095. This value is calculated by multiplying the success probability for the independent experiments. Since the *P_TES_* value is smaller than the .1 criterion, readers should be skeptical about the reported experimental results as they relate to the theory.

**29. Seifried and Ulrich (2011) “Exogenous visual attention prolongs perceived duration”**

Seifried and Ulrich (2011) used the results from six experiments to identify factors that might modulate a spatial attention effect on perceived duration. Table S28 summarizes the statistics that contributed to the TES analysis and describes the estimated probability that a replication of each study would produce the same degree of success.

Experiment 1 had two groups of participants. One group judged duration of a letter while the other group first performed a letter discrimination task and then judged the duration of a presented letter. A cue could precede the letter by brightening an outline square frame around where the letter would appear. A key result was that constant error was affected by the cue, but differently for the two groups.

Experiment 2 sometimes replaced the square frames that promoted cuing with a single rectangle that ran across both possible letter positions. The key result was the absence of an interaction between cuing effects and display (squares or rectangle).

Experiment 3 used a two-rectangle paradigm to try to replicate an earlier result. One of the key results was that constant error in the valid condition was smaller than constant error in the other conditions taken together.

Experiment 4 was the same as Experiment 3 but used a different method of judging stimulus duration. One of the key results was that target duration was judged longer in the valid condition than in the invalid conditions taken together.

Experiment 5 was the same as Experiment 4 but with a different cue (filled square rather than brief brightening of a frame). One of the key results was a non-significant interaction between cue type and target duration.

Experiment 6 was similar to Experiment 2 but with a different way of measuring effects on perceived duration (constant stimuli rather than a longer judgment). A key result was an effect of cue type on the mean point of subjective equality.

Footnote 3 describes another experiment that successfully replicated a previously found result (several other Experiments published elsewhere had failed to conceptually replicate this result). A key result was that constant error was higher in the valid condition than in both invalid conditions taken together. The text is unclear whether this result applies to the general conclusions of the paper. On the one hand, the paper is investigating situations where this effect is not found; but on the other hand the paper concludes that both the current and previous effects seem to both be real, albeit in different situations.

**Table S28**. Statistical properties of the Seifried and Ulrich (2011) experimental findings.

|  | **n** | **Test**  **Statistic** | **Probability of Success** |
| --- | --- | --- | --- |
| Exp. 1 | 12, 12 | *F*(1,22)=4.62 | .510 |
| Exp. 2 (null) | 24 | *F*(1,23)=0.13 | .937 |
| Exp. 3 | 24 | *F*(1,23)=4.79 | .527 |
| Exp. 4 | 24 | *F*(1,16)=5.90 | .614 |
| Exp. 5 (null) | 34 | *F*(6,198)=2.14 | .244 |
| Exp. 6 | 12 | *t*(11)=2.67 | .619 |
| *P_TES_* |  |  | .023 |
| Footnote 3 | 30 | *F*(1,29)=5.21 | .575 |
| *P_TES2_* |  |  | .013 |

Seifried and Ulrich (2011) reported that each of the studies produced a pattern of results that supported their theoretical stance. If the theory is correct, and the population effects are as estimated by the samples, then the probability of getting six studies like these (not including footnote 3) to produce the desired pattern is *P_TES_* = .023. This value is calculated by multiplying the success probability for the independent experiments. Since the *P_TES_* value is smaller than the .1 criterion, readers should be skeptical about the reported experimental results as they relate to the theory. If the experiment in footnote 3 is included, then the success probability is *P_TES2_* = .013.

**30. Şentürk, Greenberg, and Liu (2016) “Saccade latency indexes exogenous and endogenous object-based attention”**

Şentürk et al. (2016) used the results from six experiments to conclude that object-level priority affects saccade planning. Table S29 summarizes the statistics that contributed to the TES analysis and describes the estimated probability that a replication of each study would produce the same degree of success.

Experiment 1a was a replication of the classic object-based effect with keypresses. A key result was that reaction times were faster for the same-object than for the different-object location.

Experiment 1b was similar to Experiment 1a, but measured saccade latency rather than keypress reaction time.

Experiment 2a was similar to Experiment 1a but varied the cue validity during target-present trials. A key result was a significant difference in reaction time for the cued location and the different-object location.

Experiment 2b was similar to Experiment 2a but measured saccade latency. The key result was faster saccade latency to the same-object location than to the different-object location.

Experiment 3a was similar to Experiment 1a but used an endogenous (central) cue rather than an exogenous cue. A key result was faster reaction time for the same-object than for the different-object location.

Experiment 3b was similar to Experiment 3a but measured saccade latency. A key result was a significant difference in saccade latency for the same-object and different-object locations.

**Table S29**. Statistical properties of the Şentürk et al. (2016) experimental findings.

|  | **n** | **Test**  **Statistic** | **Probability of Success** |
| --- | --- | --- | --- |
| Exp. 1a | 17 | *F*(1,16)=6.5 | .627 |
| Exp. 1b | 15 | *F*(1,14)=8.21 | .712 |
| Exp. 2a | 15 | *F*(1,14)=4.70 | .480 |
| Exp. 2b | 15 | *F*(1,14)=8.44 | .724 |
| Exp. 3a | 15 | *F*(1,14)=10.58 | .816 |
| Exp. 3b | 15 | *F*(1,14)=10.33 | .807 |
| *P_TES_* |  |  | .102 |

Estimating success probabilities across experiments is a bit complicated because a small number of participants contributed to multiple experiments, which means the samples were not quite random and the results are not independent. There is not enough information to account for these dependencies, so we followed the lead of the original authors and treated the statistical outcomes as if they were from independent samples.

Şentürk et al. (2016) reported that each of the studies produced a pattern of results that supported their theoretical stance. If the theory is correct, and the population effects are as estimated by the samples, then the probability of getting six studies like these to produce the desired pattern is *P_TES_* = .102. This value is calculated by multiplying the success probability for the independent experiments. Since the *P_TES_* value is larger than the .1 criterion, readers do not need to be skeptical about the reported experimental results as they relate to the theory. We should note that each experiment included additional successful tests, so the estimates here should be considered as overestimates of the true success probabilities.

**31. Shomstein and Behrmann (2008) “Object-based attention: Strength of object representation and attentional guidance”**

Shomstein and Behrmann (2008) used the results from seven experiments to conclude that object-based effects are due to the strength of object representations and probabilistic contingencies in the environment. Table S30 summarizes the statistics that contributed to the TES analysis and describes the estimated probability that a replication of each study would produce the same degree of success.

Experiment 1 was in two parts that varied the preview time for a standard two-rectangles paradigm. Experiment 1a used a preview time of 1000 ms, and a key result was a significant reaction time advantage for the cued object compared to the uncued object. Experiment 1b used a preview time of only 200 ms, and a key result was a non-significant main effect of object. The text does not provide precise statistics for Experiment 1b, so we estimate the success probability as the compliment of the most favorable Type I error rate.

Experiment 2 was similar to Experiment 1b but removed a probabilistic imbalance of target appearance that was used in Experiment 1. A key result was a significant effect of object.

Experiment 3 used more salient objects (filled rather than outline rectangles) to see if object effects could overcome probabilistic imbalance. A between subjects design was used for different preview durations. A key result was an interaction between preview duration and object condition.

Experiment 4 further enhanced the objects by making them different colors. The key result was a significant interaction between probability and object condition.

Experiment 5 used a target detection task rather than the discrimination task that was used in the previous experiments. Preview duration was again a between subjects factor. A key result for Experiment 5a (preview of 1000 ms) was a significant main effect of validity. For Experiment 5b (preview of 200 ms) a key result was a significant main effect of probability.

**Table S30**. Statistical properties of the Shomstein and Behrmann (2008) experimental findings.

|  | **n** | **Test**  **Statistic** | **Probability of Success** |
| --- | --- | --- | --- |
| Exp. 1a | 15 | *F*(1,14)=6.73 | .626 |
| Exp. 1b (null) | 15 | -- | .950 |
| Exp. 2 | 19 | *F*(1,18)=5.6 | .573 |
| Exp. 3 | 15, 16 | *F*(1,29)=6.67 | .681 |
| Exp. 4 | 15 | *F*(1,14)=5.15 | .515 |
| Exp. 5a | 15 | *F*(1,14)=5.23 | .521 |
| Exp. 5b | 15 | *F*(1,14)=16.82 | .950 |
| *P_TES_* |  |  | .059 |

Shomstein and Behrmann (2008) reported that each of the studies produced a pattern of results that supported their theoretical stance. If the theory is correct, and the population effects are as estimated by the samples, then the probability of getting seven studies like these to produce the desired pattern is *P_TES_* = .059. This value is calculated by multiplying the success probability for the independent experiments. Since the *P_TES_* value is smaller than the .1 criterion, readers should be skeptical about the reported experimental results as they relate to the theory.

**32. Shomstein and Johnson (2013) “Shaping attention with reward: Effects of reward on space- and object-based selection”**

Shomstein and Johnson (2013) used the results from six experiments to conclude that guidance of selective attention is flexible and adjusts with external nonsensory reward-based factors. Table S31 summarizes the statistics that contributed to the TES analysis and describes the estimated probability that a replication of each study would produce the same degree of success.

Experiment 1a demonstrated that space- and object-based effects could be produced in a modified two-rectangles paradigm. The conclusions were based on seven significant tests and one non-significant test. We estimated the success rate for all of these tests by generating simulated data that drew random samples from populations having the means, standard deviation, and correlation present in the original data.

Experiment 1b had a between subjects design with separate analyses applied to each group. The experiment was very similar to Experiment 1a, but participants either had no-reward or a reward that favored the different object. The no-reward group (Experiment 1bi) had the standard same-object advantage. The reward group (Experiment 1bii) showed a reverse effect.

Supplemental material in Shomstein and Johnson (2013) describes a replication of the reward group, which Table S31 refers to as Experiment 1c. It showed the same reversed object-based effect.

Experiment 2 used a random reward structure, and the key finding was the absence of a significant object-based effect.

Supplemental material in Shomstein and Johnson (2013) describes a replication of Experiment 2, which Table S31 refers to as Experiment 2b. It reported the same null result. No statistics are provided, so we give the highest possible success rate for a null result: the complement of the Type I error rate.

**Table S31**. Statistical properties of the Shomstein and Johnson (2013) experimental findings.

|  | **n** | **Test**  **Statistic** | **Probability of Success** |
| --- | --- | --- | --- |
| Exp. 1a | 13 | Multiple tests | .353 |
| Exp. 1bi | 11 | *F*(1,10)=4.36 | .467 |
| Exp. 1bii | 14 | *F*(1,13)=7.65 | .673 |
| Exp. 1c | 14 | *F*(1,13)=5.13 | .505 |
| Exp. 2 (null) | 10 | *F*(1,9)=3.0 | .706 |
| Exp. 2b (null) | 14 | -- | .950 |
| *P_TES_* |  |  | .074 |

Shomstein and Johnson (2013) reported that each of the studies produced a pattern of results that supported their theoretical stance. If the theory is correct, and the population effects are as estimated by the samples, then the probability of getting six studies like these to produce the desired pattern is *P_TES_* = .074. This value is calculated by multiplying the success probability for the independent experiments. Since the *P_TES_* value is smaller than the .1 criterion, readers should be skeptical about the reported experimental results as they relate to the theory.

**33. Shomstein and Yantis (2002) “Object-based attention: Sensory modulation or priority setting?”**

Shomstein and Yantis (2002) used the results from five experiments to conclude that object-based selection is derived from an object-specific prioritization strategy rather than modulation of sensory representations. Table S32 summarizes the statistics that contributed to the TES analysis and describes the estimated probability that a replication of each study would produce the same degree of success.

Experiment 1 used a flanker task where the flanking elements could either be in the same object as a central target or in a different object. The key finding was a non-significant interaction between compatibility of target and flankers and the object condition. The text does not give a specific test statistic, so we give the highest possible success rate for a null result.

Experiment 2 was similar to Experiment 1 but increased the distance between the target and flankers. Again, the key result was a null result for the interaction of compatibility and object condition.

Experiment 3 was similar but varied target-flanker distance within subjects. One of the key test results was a significant interaction of compatibility and distance, which demonstrated that the experiment could detect effects on flanker distance.

Experiment 4 changed the colors of the rectangles (to make them more distinctive) and varied the distance between the target and the flankers. The key findings were null results for four different (orthogonal ANOVA) tests. The probability of success was estimated as the complement of the Type I error rate to the fourth power.

Experiment 5 induced a narrow attentive state by briefly flashing an uninformative cue before presenting the target and flankers. A key result was a significant effect of relation (same or different object for target and flankers) in error rates.

**Table S32**. Statistical properties of the Shomstein and Yantis (2002) experimental findings.

|  | **n** | **Test**  **Statistic** | **Probability of Success** |
| --- | --- | --- | --- |
| Exp. 1 (null) | 16 | *F*(1,15)<1 | .950 |
| Exp. 2 (null) | 16 | *F*(1,15)<1 | .950 |
| Exp. 3 | 19 | *F*(1,18)=4.5 | .485 |
| Exp. 4 (null) | 11 | Multiple tests | .815 |
| Exp. 5 | 20 | *F*(1,19)=5.46 | .567 |
| *P_TES_* |  |  | .202 |

Shomstein and Yantis (2002) reported that each of the studies produced a pattern of results that supported their theoretical stance. If the theory is correct, and the population effects are as estimated by the samples, then the probability of getting five studies like these to produce the desired pattern is *P_TES_* = .202. This value is calculated by multiplying the success probability for the independent experiments. Since the *P_TES_* value is larger than the .1 criterion, readers do not need to be skeptical about the reported experimental results as they relate to the theory. We should note that there are several tests in Shomstein and Yantis (2002) where the reported *p*-value does not match the given degrees of freedom and test statistic value.

**34. Smith, Ball, Swalwell, and Schenk (2016) “Object-based attentional facilitation and inhibition are neuropsychologically dissociated”**

Smith et al. (2016) used the results from four experiments to compare object-centered attentional facilitation and inhibition in a patient, D. F. (see also de-Wit *et al.*, 2009) with visual form agnosia to non-patients. Table S33 summarizes the statistics that contributed to the TES analysis and describes the estimated probability that a replication of each study would produce the same degree of success.

Experiment 1 cued one of three objects that then moved in a coherent fashion before a target appeared on one of the objects. Results were analyzed separately for D. F. and for a set of control participants. A critical test for D. F. (Experiment 1a in Table S33) was a significant slowing of reaction time to the target for valid-object versus invalid-object trials. Here, the sample size refers to the number of trials in the different conditions. For the control participants (Experiment 1b in Table S33), a key result was a significant location inhibitory cuing effect.

Experiment 2 was a version of the two-rectangles method with different stimulus onset asynchronies between the cue and the target. For D. F. (Experiment 2a) a key result was a null interaction of stimulus onset asynchrony and validity. The text does not indicate the number of trials that contribute to this analysis, so it is not possible to calculate an estimated success probability from the given test statistic. Instead, we use the maximum possible value (the complement of the Type I error rate). For control patients (Experiment 2b), a key result was a significant within-object inhibitory cuing effect at stimulus onset asynchrony of 1200 ms.

Experiment 3 was the same as Experiment 2 except that (to provide more powerful cues to objecthood) it used filled white rectangles instead of outline rectangles. An analysis of D. F.’s data (Experiment 3a) showed a significant difference between invalid within trials and invalid between trials. The control participants (Experiment 3b) showed a within-object advantage for a stimulus onset asynchrony of 200 ms.

Experiment 4 was similar to Experiment 3 but used a detection task rather than a discrimination task. For D. F. (Experiment 4a), a key result was a significant difference between valid and invalid-between trials, which indicated the presence of a spatial inhibitory cuing effect. For the control patients (Experiment 4b), a key result was a significant, one-tailed, difference for invalid-within and invalid-between trials, which indicated a within-object inhibitory cuing effect.

Smith et al. (2016) reported that each of the studies produced a pattern of results that supported their theoretical stance. If the theory is correct, and the population effects are as estimated by the samples, then the probability of getting four studies like these to produce the desired pattern is *P_TES_* = .015. This value is calculated by multiplying the success probability for the independent tests. Since the *P_TES_* value is smaller than the .1 criterion, readers should be skeptical about the reported experimental results as they relate to the theory. We should note that Smith et al. (2016) based their conclusions on many additional tests, but we cannot compute the success probability estimate for all tests because the outcomes are related. The *P_TES_* value in Table S33 should be considered an overestimate of success probability for experiments like these.

**Table S33**. Statistical properties of the Smith et al. (2016) experimental findings.

|  | **n** | **Test**  **Statistic** | **Probability of Success** |
| --- | --- | --- | --- |
| Exp. 1a | 107, 108 | *t*(213)=2.44 | .677 |
| Exp. 1b | 10 | *t*(9)=2.44 | .513 |
| Exp. 2a (null) | -- | *--* | .950 |
| Exp. 2b | 8 | *t*(7)=2.6 | .513 |
| Exp. 3a | 83, 83 | *t*(164)=2.02 | .516 |
| Exp. 3b | 8 | *t*(7)=2.84 | .584 |
| Exp. 4a | 52, 53 | *t*(103)=2.34 | .634 |
| Exp. 4b | 7 | *t*(6)=2.07 | .480 |
| *P_TES_* |  |  | .015 |

**35. Vecera and Behrmann (1997) “Spatial attention does not require preattentive grouping”**

Vecera and Behrmann (1997) used the results from six experiments to conclude that preattentive grouping and spatial attention are isolated processes that together guide attentional selection of visual stimuli. Table S34 summarizes the statistics that contributed to the TES analysis and describes the estimated probability that a replication of each study would produce the same degree of success.

Experiment 1 investigated the spatial attention properties of a patient with apperceptive agnosia (impaired shape processing). The patient responded to the appearance of a target that appeared in one of two peripheral boxes after a cue (exogenous or endogenous). One key result (labeled as Experiment 1a in Table S34) was a significant difference in reaction time for cue type. The sample size listed in Table S34 refers to the number of trials in the conditions. A second key result (labeled as Experiment 1b) is a non-significant interaction between target location and cue type. The text does not give a precise test statistic, so we assigned the maximum possible success probability for a null result (the complement of Type I error).

Experiment 2 tested the patient with a version of the two-rectangles method. Separate analyses were applied to results in a first (Experiment 2a) and a second (Experiment 2b) session; both of which showed a non-significant result for same versus different object cuing.

Experiment 3 was similar to Experiment 2, but added a “near” condition where the rectangles were close to fixation. One key result (Experiment 3a) was a significant one-tailed test for same versus different objects in the near condition. A second key result (Experiment 3b) was a non-significant difference between same versus different objects in the equidistant condition. The text does not give a precise test statistic, so we assigned the maximum possible success probability for a null result (the complement of Type I error).

Experiment 4 asked the patient to report the orientation of rectangles. There were no statistical analyses because the patient was 100% correct.

Experiment 5 asked the patient to perform a variety of grouping tasks. Estimated power for several of the tasks was quite high (essentially 1.0), but for a good continuation task performance was rather poor. A key statistical result was performance below chance.

Experiment 6 tested the patient’s symmetry perception in two ways. On the first task (Experiment 6a), the key result was a non-significant deviation from chance performance. The key result for the second task (Experiment 6b) was a null result for a response bias (to reporting red shapes as figure). Note that the reported χ^2^ does not match the reported frequencies for Experiment 6b. Table S34 reports the correct χ^2^ value.

**Table S34**. Statistical properties of the Vecera and Behrmann (1997) experimental findings.

|  | **n** | **Test**  **Statistic** | **Probability of Success** |
| --- | --- | --- | --- |
| Exp. 1a | 359, 359 | *F*(1,716)=4.47 | .559 |
| Exp. 1b (null) | 359, 359 | *F*(1,716)<1 | .950 |
| Exp. 2a (null) | 30, 30 | *t*(58)=0.228 | .923 |
| Exp. 2b (null) | 32, 33 | *t*(63)=0.46 | .884 |
| Exp. 3a | 56, 56 | *t*(110)=-1.89 | .587 |
| Exp. 3b (null) | -- | -- | .950 |
| Exp. 5 | 32 | χ^2^(1)=4.88 | .551 |
| Exp. 6a (null) | 64 | χ^2^(1)=1.00 | .832 |
| Exp. 6b (null) | 64 | χ^2^(1)=1.56 | .764 |
| *P_TES_* |  |  | .085 |

Vecera and Behrmann (1997) reported that each of the studies produced a pattern of results that supported their theoretical stance. If the theory is correct, and the population effects are as estimated by the samples, then the probability of getting six studies like these to produce the desired pattern is *P_TES_* = .085. This value is calculated by multiplying the success probability for the independent tests. Since the *P_TES_* value is smaller than the .1 criterion, readers should be skeptical about the reported experimental results as they relate to the theory.

**36. Yeari and Goldsmith (2010) “Is object-based attention mandatory? Strategic control over mode of attention”**

Yeari and Goldsmith (2010) used the results from five experiments to conclude that object-based attention seems to be a default mode that can be overridden by strategic control. Table S35 summarizes the statistics that contributed to the TES analysis and describes the estimated probability that a replication of each study would produce the same degree of success.

Experiment 1a investigated the ability of participants to avoid attentional influence from perceptual grouping. A non-informative central arrow cue pointed to one of two peripheral elements where a target might appear. A key result was a lower error rate for same-group targets than for different-group targets.

Experiment 1b was similar to Experiment 1a, but the central cue was informative (cued the target on 80% of trials). The weakest key result (one-tailed test) was a significantly faster reaction time for different-objects compared to same-objects for a stimulus onset asynchrony of 300 ms.

Experiment 2a used a non-informative cue with a connected-object. A key finding was a significant object-effect for error rates.

Experiment 2b was similar to Experiment 2a but used an informative cue. A key result was that responses to same-object targets were slower than responses to different-object targets.

Footnote 3 describes a control experiment that ruled out a salience “pop-out” explanation. The key result was a null same-object advantage. The text does not give a precise test statistic, so we used the largest possible success probability (the complement of the Type I error rate).

**Table S35**. Statistical properties of the Yeari and Goldsmith (2010) experimental findings.

|  | **n** | **Test**  **Statistic** | **Probability of Success** |
| --- | --- | --- | --- |
| Exp. 1a | 20 | *F*(1,19)=5.0 | .531 |
| Exp. 1b | 20 | *t*(19)=1.8 | .508 |
| Exp. 2a | 15 | *F*(1,14)=5.6 | .549 |
| Exp. 2b | 16 | *F*(1,15)=6.5 | .619 |
| Footnote 3 | 14 | *F*(1,13)<1 | .950 |
| *P_TES_* |  |  | .087 |

Yeari and Goldsmith (2010) reported that each of the studies produced a pattern of results that supported their theoretical stance. If the theory is correct, and the population effects are as estimated by the samples, then the probability of getting five studies like these to produce the desired pattern is *P_TES_* = .087. This value is calculated by multiplying the success probability for the independent tests. Since the *P_TES_* value is smaller than the .1 criterion, readers should be skeptical about the reported experimental results as they relate to the theory. We should note that the conclusions depended on many other test outcomes within and between experiments, so the *P_TES_* in Table S35 almost surely overestimates the true probability of success for all tests.

**37. Yin, Xu, Duan, and Shen (2018) “Object-based attention on social units: Visual selection of hands performing a social interaction”**

Yin et al. (2018) used the results from six experiments to conclude that object-based attention is constrained by top-down information. Table S36 summarizes the statistics that contributed to the TES analysis and describes the estimated probability that a replication of each study would produce the same degree of success.

Experiment 1 was a variation of the two-rectangles paradigm where the rectangles were replaced by images of hands that sometimes were arranged as in a traditional handshake and sometimes reversed. The analysis was quite complicated and involved more than 7 tests. Many of those tests have high power estimates, but a comparison of the cueing effects for the reversed vertical and handshake vertical conditions have modest power of around 0.75 (even when considering the use of a non-standard significance criterion of 0.057 rather than 0.05). In addition, a comparison of cueing effects for the reversed horizontal and handshake vertical have power of only around 0.55. Taken all together, the estimated success probability for the full set of results is fairly low.

The supplemental material in Yin et al. (2018) included an additional four experimental results that supported the conclusions. Experiment S1a was the same as Experiment 1, but used only the handshake condition. A key result was a significant difference in reaction time between the invalid horizontal-hand and invalid vertical-hand conditions. Following the procedure in Yin et al. (2018) the significance criterion was Bonferroni corrected to be 0.05/3. We note that the *p*-value reported by Yin et al. (2018) was calculated by multiplying the *p*-value from the *t*-test by the number of tests (3).

Experiment S1b was the same as Experiment S1a except that the hand images did not complete a handshake. A key result was a non-significant difference between the invalid horizontal-hand and invalid vertical-hand conditions.

Experiment S2 was similar to Experiment 1 except that the reversed-hand condition was replaced by the reversed-handshake condition (both hands handshaking upside down). A key result was a significant interaction between display type and validity.

Experiment S3 was similar to Experiment 1 except that the entire display was rotated 90 degrees. The weakest key result was a smaller cue effect for a handshake in the horizontal alignment compared to the vertical alignment.

**Table S36**. Statistical properties of the Yin et al. (2018) experimental findings.

|  | **n** | **Test**  **Statistic** | **Probability of Success** |
| --- | --- | --- | --- |
| Exp. 1 | 20 | Multiple tests | .429 |
| Exp. 2 (null) | 20 | *F*(2,38)=0.52 | .871 |
| Exp. S1a | 20 | *t*(19)=2.94 | .586 |
| Exp. S1b (null) | 20 | *t*(19)=0.26 | .943 |
| Exp. S2 | 20 | *F*(2, 38)=4.40 | .725 |
| Exp. S3 | 20 | *t*(19)=2.47 | .614 |
| *P_TES_* |  |  | .092 |

Yin et al. (2018) reported that each of the studies produced a pattern of results that supported their theoretical stance. If the theory is correct, and the population effects are as estimated by the samples, then the probability of getting six studies like these to produce the desired pattern is *P_TES_* = .092. This value is calculated by multiplying the success probability for the independent tests. Since the *P_TES_* value is smaller than the .1 criterion, readers should be skeptical about the reported experimental results as they relate to the theory.

**38. Zemel, Behrmann, Mozer, and Bavelier (2002) “Experience-dependent perceptual grouping and object-based attention”**

Zemel et al. (2002) used the results from four experiments to conclude that the perceptual system can flexibly apply object benefits as a function of experience. Table S37 summarizes the statistics that contributed to the TES analysis and describes the estimated probability that a replication of each study would produce the same degree of success.

Experiment 1 had participants make speeded same-different judgments about features on the ends of shapes that could be perceived as connected or disconnected. The weakest key result was a significant three-way interaction between experiment block, group (connected or disconnected), and feature location.

Experiment 2 used new stimuli that made the feature ends seem connected or disconnected. The key result was a significant three-way interaction between feature location, display type, and epoch.

Experiment 3 was similar to Experiment 2, but used different stimuli. The weakest key result was a non-significant interaction between feature location and display type.

Experiment 4 was similar to Experiment 3 but added an initial pretest block and changed where the features were located. A key result was that diagonal features were processed faster than vertical features; significance was claimed for *p*<0.065.

**Table S37**. Statistical properties of the Zemel et al. (2002) experimental findings.

|  | **n** | **Test**  **Statistic** | **Probability of Success** |
| --- | --- | --- | --- |
| Exp. 1 | 30 | *F*(2,56)=3.32 | .606 |
| Exp. 2 | 18 | *F*(1,17)=6.03 | .599 |
| Exp. 3 (null) | 24 | *F*(1,23)=0.23 | .927 |
| Exp. 4 | 24 | *F*(1,23)=3.80 | .488 |
| *P_TES_* |  |  | .164 |

Zemel et al. (2002) reported that each of the studies produced a pattern of results that supported their theoretical stance. If the theory is correct, and the population effects are as estimated by the samples, then the probability of getting four studies like these to produce the desired pattern is *P_TES_* = .164. This value is calculated by multiplying the success probability for the independent tests. Since the *P_TES_* value is larger than the .1 criterion, readers do not need to be skeptical about the reported experimental results as they relate to the theory. However, Zemel et al. (2002) based their conclusions on additional tests, but we do not have enough information to include them all. Thus, *P_TES_* = .164 should be considered an overestimate of the true success probability.

**39. Zhao, Kong, and Wang (2013) “Attentional spreading in object-based attention: The roles of target-object integration and target presentation time”**

Zhao et al. (2013) used the results from five experiments to conclude that object-based attention is due to attentional spreading. Table S38 summarizes the statistics that contributed to the TES analysis and describes the estimated probability that a replication of each study would produce the same degree of success.

Experiment 1a used crossed rectangles with a central target letter and flankers that could be on the same or different object (rectangle). The weakest relevant result was a significant main effect of compatibility for error rates.

Experiment 1b was similar to Experiment 1a but used different presentation times for the target and flankers. The weakest relevant result was a significant main effect of compatibility for error rates.

Experiment 2a was similar to Experiment 1b but with still different presentation times. A key result was a significant main effect of relation for reaction times.

Experiment 2b was also similar to Experiment 1b but with still different presentation times (Experiments 2a and 2b presented the rectangles for equal durations, thus controlling one potential effect).

Experiment 3 used an entirely new type of stimulus and task. Participants classified a central cutout shape in contexts where flanking cutout shapes could be on the same object or on different objects. A key result was a significant effect of relation.

**Table S38**. Statistical properties of the Zhao et al. (2013) experimental findings.

|  | **n** | **Test**  **Statistic** | **Probability of Success** |
| --- | --- | --- | --- |
| Exp. 1a | 17 | *F*(1,16)=7.65 | .696 |
| Exp. 1b | 17 | *F*(1,16)=6.6 | .632 |
| Exp. 2a | 17 | *F*(1,16)=4.92 | .510 |
| Exp. 2b | 18 | *t*(17)=2.16 | .495 |
| Exp. 3 | 17, 17 | *F*(1,32)=7.29 | .725 |
| *P_TES_* |  |  | .080 |

Zhao et al. (2013) reported that each of the studies produced a pattern of results that supported their theoretical stance. If the theory is correct, and the population effects are as estimated by the samples, then the probability of getting five studies like these to produce the desired pattern is *P_TES_* = .080. This value is calculated by multiplying the success probability for the independent tests. Since the *P_TES_* value is smaller than the .1 criterion, readers should be skeptical about the reported experimental results as they relate to the theory.

**40. Power analysis for future studies of object-based attention: The possible impact of varying the number of trials**

In the conclusion section of the main text, we provide a power analysis that suggests future studies wanting to demonstrate the simplest object-based attention effect (a significant difference in response time for invalid same and invalid different trials) need to use at least *n*=324 observers to have 90% power.

In the experiment in the main text that was used for this power analysis, each mean value for each observer was computed from 18 trials (recall that 80% of the 180 experimental trials were for the valid condition). Power can be influenced by the number of trials (Francis, 2019; Baker et al., 2021) because the mean value computed for each observer varies, in part, due to sampling variability across trials. As the number of trials increases the variability of the mean for each observer decreases, and this has an influence on computations of variability across observers. Thus, the variability across observers can be reduced by increasing the number of trials per observer. However, there is a limit to this reduction because for very large trial numbers the variability within each observer is very small relative to the variability across observers.

We can compute the impact of increasing the number of trials on the variability across participants. This is most easily done by computing the difference between mean invalid same and mean invalid different response times for each observer. Based on the data from our experiment, we estimate that the mean difference across observers is 14 milliseconds, the standard deviations across the *k*=18 trials within an observer is around *S_w_*=216 milliseconds and the observed standard deviation of differences across observers is around *S_s_*=77.5 milliseconds. By the variance sum law, the standard deviation across observers that is not due to variability within an observer is estimated to be

$S_{b}=\sqrt{S_{s}^{2}-\frac{S_{w}^{2}}{k}}\approx$58.4

If we imagine an experiment with more trials (*k*>18), then a new calculation for the standard deviation of differences across observers will be

$${\text{new}S}_{s}=\sqrt{S_{b}^{2}+\frac{S_{w}^{2}}{\text{new}k}}$$

If we doubled the number of trials (new*k*=36), then new*S_s_*=68.6 milliseconds, and then 90% power can be achieved with *n*=255 observers. If we halved the number of trials (new*k*=9), then new*S_s_*=92.7 milliseconds, and 90% power will require *n*=463 observers. If new*k* is very large, new*S_s_* $\approx$*S_b_* and then 90% power can be achieved with *n*=185 observers. Given the information available, this is the minimum possible sample size to have 90% power to detect a significant difference between the invalid same and invalid different conditions. We note that this sample size is much larger than all experiments in Table 4 of the main text.

**References not in main text**

Baker, D. H., Vilidaite, G., Lygo, F. A., Smith, A. K., Flack, T. R., Gouws, A. D., & Andrews, T. J. (2021). Power contours: Optimising sample size and precision in experimental psychology and human neuroscience. Psychological Methods, 26(3), 295-314. http://dx.doi.org/10.1037/met0000337

Behrmann, M., Zemel, R. S. & Mozer, M. C. (1998). Object-based attention and occlusion: Evidence from normal participants and a computational model. *Journal of Experimental Psychology: Human Perception & Performance*, *24*(4), 1011-1036.

Chou, W.-L. & Yeah, S.-L. (2011). Subliminal spatial cues capture attention and strengthen between-object link. *Consciousness & Cognition, 20*, 1265-1271.

Crawford, J. R. & Garthwaite, P. H. (2007) Comparison of a single case to a control or normative sample in neuropsychology: Development of a Bayesian approach. *Cognitive Neuropsychology*, *24*(4), 343-372, DOI: 10.1080/02643290701290146

Davis, G. & Holmes, A. (2005) Reversal of object-based benefits in visual attention, *Visual Cognition*, *12*(5), 817-846, DOI: 10.1080/13506280444000247

Donovan, I., Pratt, J. & Shomstein, S. (2017). Spatial attention is necessary for object-based attention: Evidence from temporal-order judgments. *Attention, Perception & Psychophysics*, *79*, 753-664.

Francis, G. (2019). Hypothesis testing reconsidered. In J. Enns (Ed.) *Cambridge Elements of Perception*, https://doi.org/10.1017/9781108582995

Greenberg, A. S., Rosen, M., Cutrone, E. & Behrmann, M. (2015). The effects of visual search efficiency on object-based attention. *Attention, Perception & Psychophysics*, *77*, 1544-1577.

Ho, M.-C. & Yeh, S.-L. (2009). Effect of instantaneous object input and past experience on object-based attention. *Acta Psychologica*, *132*, 31-39.

Lazareva, O. F., Vecera, S. P., Levin, J. & Wasserman, E. A. (2005). Object discrimination by pigeons: Effects of object color and shape. *Behavioural Processes*, *69*, 17-31.

Lazareva, O. F., Vecera, S. P. & Wasserman, E. A. (2006). Object discrimination in pigeons: Effects of local and global cues. *Vision Research*, *46*, 1361-1374.

Lou, C. & Proctor, R. W. (2016). Perceptual grouping of objects occupied by target and flankers affects target-flanker interference. *Attention, Perception & Psychophysics*, *78*, 251-263.

Mapelli, D., Cherubini, P. & Umiltà, C. (2002). Attending to objects: Costs or benefits? *Acta Psycholgica*, *109*, 57-74.

Nuijten, M. B., Hartgerink, C. H. J., van Assen, M. A. L. M., Epskamp, S., & Wicherts, J. M. (2015). The prevalence of statistical reporting errors in psychology (1985-2013). *Behavior Research Methods*, *48* (4), 1205-1226. DOI: 10.3758/s13428-015-0664-2
